# Preparation of antibacterial cellulose of non-woven cotton fabric treated with curcumin/shellac based on polyvinyl alcohol/sodium caseinate blends for potential packaging purposes

**DOI:** 10.1038/s41598-025-97975-4

**Published:** 2025-05-02

**Authors:** Salah A. A. Mohamed, Mohamed El-Sakhawy, Hesham M. Fahmy

**Affiliations:** 1 Packing and Packaging Materials Department, National Research Centre, 33 El Bohouth St., Dokki, P.O. 12622, Giza, Egypt; 2 Cellulose & Paper Department, National Research Centre, 33 El Bohouth St., Dokki, P.O. 12622, Giza, Egypt; 3 Textile Research and Technology Institute, National Research Center, Dokki, P.O. 12622, Giza, Egypt

**Keywords:** Packaging films, Non-woven cotton fabric, Polyvinyl alcohol, Sodium caseinate, Curcumin-shellac, Biomaterials, Biochemistry, Chemistry, Materials science, Nanoscience and technology

## Abstract

Polysaccharides are renewable resources and can be used as alternative packaging materials that can serve as petroleum-based polymers. Non-woven fabrics are widely used in packaging because of their durability, ease of storage, and lightweight nature. Combining advantages of these materials offers benefits such as easy recyclability, molding simplicity, strong tear resistance and a compact design. In this research, novel antimicrobial packaging materials were fabricated by treating of non-woven cotton (NWC) fabric samples with various formulations containing polyvinyl alcohol (PVA) and sodium caseinate (SC) followed by treatment with curcumin and/or shellac followed by cross-linking via an aqueous solution of acetic acid (AC). Factors influencing formation of these formulations films were studied. The results obtained revealed that the optimal conditions for preparing crosslinked PVA/SC film with good performance properties are: PVA/SC weight ratio, 25/75 respectively; AC concentration, 0.25%; and immersion time, 10 min. The chemical structure of the PVA/SC film under optimum conditions was confirmed through FTIR analysis. Toxicity evaluations indicated that casein exhibits minimal toxicity to brine shrimp, even at high concentrations. Shellac was found to be relatively safe (10 mg/L had 0.53% mortality), PVA displayed moderate toxicity. Curcumin is relatively non-toxic at lower concentrations, and the composite D12 (PVA/SC/SH/Ag-NPs) film recorded a mortality rate of 10.3% (low toxicity) at a concentration of 10 mg/L. To enhance the antimicrobial properties, bio-additives such as curcumin, shellac, silver nanoparticles, or their binary admixtures were incorporated into the film formulations. Among the antimicrobial PVA/SC (25/75) films, the film containing curcumin/shellac at a weight ratio of 0.075/10 exhibited the best performance properties. Applying the aforementioned PVA/SC/curcumin/shellac formulation to NWC fabric sample resulted in: (a) Reduced swelling properties accompanied by an increasing in gel fraction of the treated fabric (b) Improved antimicrobial activity of the treated fabric against Gram-positive bacteria (*Staphylococcus aureus*), Gram-negative bacteria (*Escherichia coli*), pathogenic yeast (*Candida albicans*), and filamentous fungus: (*Aspergillus niger*), (c) Decreased air permeability along with an increased tensile strength, Young’s and burst strength of the treated fabric, and (d) The best water vapor transmission rate compared to other treated formulations. The maximum hydrophobicity results increased the contact angle of NWC by 1219.28 and 1461.13%, respectively, after the addition of PVA/SC as in (NW1, 105.05°) and PVA/SC/CUR/SH as in (NW9, 124.31°) as compared to untreated non-woven. The successful incorporation of PVA/SC or PVA/SC/Cur/SH into NWC was evidenced by the X-ray analysis, which showed decreased crystallinity. Additionally, TGA analysis indicated that the non-woven cellulose’s thermal stability was enhanced by the addition of PVA/SC/CUR-SH. The chemical structure of the treated fabric was confirmed through FTIR analysis, while its morphology was investigated using SEM analysis. These findings support the potential application of the PVA/SC/curcumin/shellac-treated fabric as a packaging material.

##  Introduction

Polysaccharides such as chitosan, cellulose, non-woven textiles, carboxymethyl cellulose, cyclodextrins and hydroxypropyl methylcellulose^1–4^ as well as natural proteins such as casein, zein, soy protein, gelatin, whey and casein protein are renewable sources. Based on an oxidized corn starch-based nonionic biopolymer and gelatin, the study introduces a novel class of biomass-based multifunctional antibacterial packaging films that are both environmentally friendly and biodegradable^5,6^. A sustainable one-pot approach was used to create two novel cellulose-based nonionic antibacterial polymers with many terminal indole groups^7,8^. They are used as alternative packaging materials or as additives in packaging materials to replace petrol-based polymers^9–12^.

Non-woven fabrics are becoming increasingly popular in packaging because of their durability, simplicity of storage, and lightweight nature. The benefits of packing with non-woven materials are easy recyclability, molding simplicity, strong durability against tearing, and a compact design^13^. Non-woven fabric is a material that resembles cloth and is composed of long (continuous long) and staple (short) fibers that have been joined by solvent, heat, mechanical, or chemical treatment. In the textile production sector, the phrase refers to materials that are neither knitted nor woven, such as felt^14^. In general, non-woven textiles are sheet or web structures that are joined by mechanical, thermal, or chemical bonding of entangled fibers or filaments (as well as perforating films). These are porous sheets that can be flat or tufted and are created straight from individual fibers, melted plastic or plastic film. They don’t involve turning the fibers into yarn, nor are they created by knitting or weaving. Non-woven fabrics usually contain a certain amount of oil-based ingredients and recycled textiles^15^. Another study introduced non-woven fabric for packaging. In that study, the cost-effectiveness of non-woven fabrics was evaluated^16^. Nowadays, many commercial websites on the internet promote the use of non-woven-based packaging materials. The strength of the material required for the particular usage determines the percentage of recycled fabrics. Additionally, with the right care and resources, certain non-woven textiles can be recycled after use. Because of this, some people believe that non-woven fabric is more environmentally friendly for specific uses, particularly in sectors and businesses such as healthcare, education, nursing homes, and upscale lodging where single-use or throwaway goods are crucial^14^. Moreover, composites can be created based on textile waste that possesses sufficient mechanical, acoustical, and thermal qualities for use in the construction or automotive industries. Three recycled non-woven wastes—cotton, polyester, and cotton/polyester blend—are used for this purpose and mixed with epoxy resin. The vacuum infusion technique is used to manufacture the composite panels^17^. The prospect of altering a commercial viscose non-woven fabric used in the hygiene sector is discussed. The non-woven fabric was dyed brown, soaked in KNO_3_ solution, and then coated with PLA solution as part of the modification^18^.

Certain non-woven textiles are not strong enough unless they are reinforced or densified by a backing. Non-woven materials have emerged as a substitute for polyurethane foam in recent years^15^. In a novel study, non-woven fabric was treated with waste plastic bottles for treating domestic wastewater^19^. In another study, non-woven cotton fabric samples were treated with multiple layers of hyaluronan and chitosan using layer-by-layer assembly techniques for fabricating novel wound dressings^20^.

Casein is the primary proteinaceous component of milk. Casein micelles are highly hydrophobic and can be considered in skim milk as nanogels, bound together through hydrophobic linkages and calcium bridges^10,11,21^. Casein was previously investigated due to due to its transparent film, odorless nature, and high gas barrier properties. It is a suitable option for edible film applications and food packaging because of these factors. However, the brittleness of casein films and their high-water vapor permeability restrict their uses. Blending casein with biodegradable polymers such as polyvinyl alcohol and a plasticizer can overcome such limitations^22^. Acetic acid can precipitate sodium caseinate (SC) from milk^23^. Casein can be precipitated using acetic acid, lemon juice, tartaric acid, or citric acid, the former being the most frequently used^24^.

In a study, Bonnaillie et al. reported that casein films could be used for food packaging applications as they have low oxygen permeability, good strength, high sensitivity to moisture, and low elasticity. Calcium-caseinate/glycerol (3:1 ratio, respectively) films were prepared and their mechanical properties were investigated. The results exhibited that at constant relative humidity (RH), these films have a good elongation at break depending on the film thickness. As RH increased, tensile strength and modulus decreased while elongation at break increased. Upon adding different concentrations of citric pectin to the above formulation, stoichiometry-dependent casein-pectin interactions were formed inside the dried films, enhancing the usefulness of pectin in casein film formulations as potential packaging materials^22^.

Polyvinyl alcohol (PVA), a water-soluble synthetic polymer, is prepared by saponification of polyvinyl acetate. The main properties of PVA are governed by its degree of polymerization and saponification. PVA can be applied industrially in textiles, paper coating, emulsifiers, artificial leather, membranes, rubber-like items, ion exchange, hydrogels, porous ceramic surfaces, etc^25–31^.

Recently, the food industry has shown increased interest in polymers due to their potential applications, which has driven the creation and use of innovative polymers that will play important roles in food products in the future^32,33^.

One such polymer that offers significant benefits for use in food applications is shellac, a well-known natural polymer with unique properties like biodegradability and biocompatibility. The technology for extracting and processing shellac has advanced, and its structure is now well known. The food industry benefits greatly from using shellac because of its characteristics. Clarification is necessary to enhance the creation of novel shellac applications in food, which depends on fabrication data from published research studies. Furthermore, there is a need to address the limitations of shellac resulting from self-polymerization and their impact on the stability of food systems based on shellac, which are rarely reported. Overall, shellac holds promise for expanded applications in the food industry^33–36^. When compared to HPMC films, the produced films exhibit better tensile strength, air permeability, biological activity, and thermal stability. Therefore, the films made of HPMC/shellac/CNTs-ZnO-NPs exhibit potential as packaging materials in the future^37^.

Curcumin, which is primarily found in turmeric as diferuloyl methane, is a naturally occurring yellow colorant that possesses a variety of advantageous characteristics, including anti-inflammatory, anti-carcinogenic, and antioxidant effects^38^. Nevertheless, these advantages are constrained by its low bioavailability due to its low stability and solubility in water (about 11 mg mL^− 1^)^39^. Although curcumin is a multipurpose active component, its low stability, bioaccessibility, freeze-dried redispersibility, and solubilization limit its utilization. Shellac/curcumin nanoparticles (S/Cur) demonstrated a high loading capacity bright spot (exceeding 70%), along with good encapsulation efficiency (minimum exceeding 85%). Hydrophobic contact and hydrogen bonding were involved in the synthesis of shellac/curcumin, and the nano confined curcumin was amorphous^40^.

Hydrogels based on natural polymers, proteins, and polysaccharides are of great importance in packaging applications. Gelation is the process of cross-linking polymer chains to form a three-dimensional network that can absorb and retain water within its structure. The polymer chains in gels can be cross-linked by covalent bonds, electrostatic forces, Van der Waals forces, hydrogen bonds, and hydrophobic interactions^41^. Because of their versatility, natural protein hydrogels are frequently employed in the food business; thus, developing hydrogels with excellent qualities. Incorporating natural bio-additives such as curcumin and shellac into PVA/SC films will enhance antimicrobial properties while improving mechanical performance and water vapor transmission rates. Additionally, it can predict that the optimized weight ratios and cross-linking conditions (e.g., acetic acid concentration, immersion time) will lead to a treated non-woven fabric exhibiting reduced swelling properties, higher gel fraction, increased hydrophobicity, and improved thermal stability.

This research work aims to establish the proper conditions to fabricate a new antibacterial packaging material by treating non-woven cotton fabric samples with PVA/SC formulations containing curcumin, shellac, silver nanoparticles, or their combinations as bio-additives followed by crosslinking with aqueous acetic acid solutions that can precipitate sodium caseinate entangled with PVA chains. This study combines natural renewable materials with durable non-woven fabrics, resulting in antimicrobial packaging materials with enhanced physical and mechanical properties. By employing PVA/SC films treated with bio-additives such as curcumin, shellac, and silver nanoparticles, this research demonstrates a novel approach to creating packaging materials that are natural, recyclable, lightweight, and resistant to tear while imparting significant antimicrobial efficacy. The study addresses research gaps in the application of bio-based films to non-woven fabric, particularly exploring the relationship between chemical composition and functional properties such as hydrophobicity, antimicrobial activity, and mechanical strength.

Table 1 is a benchmarking table in which the findings of other research articles that have similar materials or similar topics are compared with our manuscript.

**Table 1:** A benchmarking table.

Film composites	The most important results	Ref.
Caseinate solutions were blended with different concentrations of glycerol followed by drying to form flexible transparent films	The tensile strength and elastic modulus of the prepared films decreased while the moisture content increased with the increase in glycerol content.	^42^
Different concentrations of silver nanoparticles (1, 3, 5, and 7%) were added to polyvinyl alcohol and sodium caseinate blend, 60/40 respectively, (w/w). After that, the films were crosslinked via dipping in a glutaraldehyde bath.	The film containing 1% Ag-NP exhibited the highest tensile strength. The lowest moisture content was noticed in the 5% and 7% Ag-NP loaded films. Moreover, the total soluble matter amount of the films decreased with increasing amount of Ag-NPs compared with the pure crosslinked film. All films showed antibacterial properties against *Escherichia coli* and *Staphylococcus aureus*.	^43^
Genipin as a crosslinker was added to the sodium caseinate film-forming solution.	The produced films had higher Young’s modulus, tensile strength as well as lower break elongation.	^44^
Crosslinking of caseinates via the divalent cation calcium	Mechanical properties and moisture barrier were improved due to the open structure of casein coils, which tend to form intermolecular bonds through hydrogen and hydrophobic interactions, leading to a more stable structure.	^45^
Different ratios of PVA and plasticized casein (CA) were prepared to obtain different films using the solution-casting method.	The tensile strength of CA increased with increasing PVA content while the water vapour barrier of the plasticized CA was improved by blending with PVA.	^11^
Preparation of Antibacterial Cellulose of Non-Woven Cotton Fabric Treated with Curcumin/Shellac Based on Polyvinyl Alcohol/Sodium Caseinate Blends for Potential Packaging Purposes	This current research aims to establish the proper conditions to fabricate a new antibacterial packaging material by treating non-woven cotton fabric samples with polyvinyl alcohol/sodium caseinate different formulations containing curcumin, shellac, silver nanoparticles, or their combinations as bio-additives followed by crosslinking with the acetic acid aqueous solution that has the ability to precipitate sodium caseinate entangled with PVA chains.	^46^ Current work Ref: Submission ID df367c92-47e2-48a6-b2e6-aa35a4eec0c7

##  Experimental

### Materials

Non-woven 100% cotton fabric (NWC fabric), 45 g/m^2^, provided by Hebitex Co., Egypt, was used. Polyvinyl alcohol (PVA) namely Mowiol 4–80 (partially hydrolyzed, low viscosity) was obtained from Hoechst Company. Sodium caseinate (SC) and curcumin (CUR), Bio Basic Canada INC., and Shellac (SH) commercial grade were used. Silver nitrate (AgNO_3_) was obtained from Sigma-Aldrich, USA, was used to prepare the silver nano-particles. Starch, Potato starch extra pure PIOCHEM. Sodium hydroxide, acetic acid and other chemicals were of analytical grades.

### Methods

#### Preparation of PVA/SC blend solutions

The PVA solution (6%) was prepared by dissolving the PVA granules in distilled water at 85 °C/1 h with stirring. In a similar procedure, the sodium caseinate (6%) solution was prepared by heating it at 60 °C/1 h. To formulate different PVA/SC blends, various ratios of the prepared PVA and SC solutions as well as 25% of glycerol, based on the PVA/SC blend solid content (w/w), as a plasticizer were mixed with stirring followed by sonication to remove the air bubbles, casting, and drying at 45 ^o^C^47^.

#### Preparation of crosslinked PVA/SC films

The solution-casting method was used to prepare the PVA/CS films. The dried films were then crosslinked by soaking in different concentrations of acetic acid solution (0.6-2%) for specific durations (3–15 min), followed by washing with distilled water and drying at 80 °C for 10 min^12^.

#### The Preparation of powdered silver nano-particles (Ag-NPs)

The silver nano-particles powder (Ag-NPs) was synthesized using native starch as reducing agent and silver nitrate as a precursor^48^. Typically, 5 g of a native starch was added to 80 ml of sodium hydroxide solution (30% based on the starch weight). The solution temperature was then raised to 70 °C. 4.72 g of silver nitrate dissolved in 20 ml of H_2_O was added slowly to the above-mentioned alkaline solution under vigorous stirring. The reaction was then kept for 60 min at pH 12. Finally, 100 ml of absolute ethanol was slowly added to the aforementioned solution to precipitate the Ag-NPs in a powder form followed by centrifugation at 4500 rpm/15 min, decantation using 80/20 ethanol/water in order to separate the unreacted components, washing again with absolute ethanol, and dried under ambient conditions.

#### Preparation of PVA/SC/bio-additives crosslinked films

To enhance the antimicrobial properties of the prepared PVA/SC films, different bio-additives namely curcumin, shellac, silver nanoparticles or their binary admixtures were added (w/w) during the PVA/SC films preparation followed by casting, drying and crosslinking with acetic acid aqueous solution as mentioned above.

#### Coating of non-woven fabric samples with the PVA/SC formulations

Non-woven cotton fabric samples were padded in different PVA/SC formulations (in the presence or absence of the above-mentioned bio-additives) at a wet pick up of 100% followed by squeezing and drying at 80 °C for 10 min. The dried samples were then crosslinked by immersion in an aqueous solution of acetic acid of a specific concentration for a particular time followed by rinsing with distilled water and drying at 80 °C for 10 min^20^.

### Testing and analysis

Each experiment was conducted at least three times to ensure accuracy. The findings presented are the average of three samples with a deviation of less than 5%, and the significance level (p) between different groups was ≤ 0.05.

Add-on was determined using the “boil-off” method^29^. The degree of swelling (SW) was assessed by steeping films or treated fabric samples in distilled water at 37 °C and pH 7 for 24 h. The samples were then removed from the water, gently wiped with a filter paper, and weighed^20,49^. The degree of swelling percentage was evaluated using Eq. 1:

1 $$SW{\text{ }}\left( \% \right){\text{ }} = {\text{ }}\left( {Wh - Wd} \right)/{\text{ }}\left( {Wd} \right){\text{ }} \times 100$$

Where Wh is the hydrated weight of the films or treated fabric samples after swelling in distilled water, and Wd is their dry weight.

The gel fraction (GF), which indicates the degree of firmness of the prepared films or treated fabric structures^20^, was calculated using Eq. 2:

2 $$GF{\text{ }}\left( \% \right){\text{ }} = {\text{ }}\left( {Wa/Wi} \right){\text{ }} \times 100$$

Where Wa is the constant weight of the dried films or treated fabric after swelling in water (pH 7, 37 °C, for 24 h), and Wi is their initial weight.

#### FTIR analysis

FTIR spectroscopy was performed using an FTIR-4700 Spectrometer (JASCO).

#### SEM imaging

SEM images were captured using SEM Model Quanta 250 FEG (Field Emission Gun) coupled with an EDX unit, with a voltage of 30 kV and magnification of 14× to 1,000,000× [38, 58–59]. The equipment was manufactured by FEI, Netherlands.

#### Tensile strength, air permeability, and WVTR

- Tensile strength (TS): Measured in the warp direction using ASTM test method D-2256-98.
- Air permeability (AP): Determined using ASTM D 737 − 96.
- Water vapor transmission rate (WVTR): Measured according to ASTM E96.

Statistical analysis Duncan’s New Multiple Range test was performed to compare the means of the treatments using the MSTAT Computer Program (MSTAT Development Team, 1989). A fully randomized block design was used for the analysis of variance. Steel and Torrie (1980) conducted a statistical analysis of the^50,51^.

#### X-Ray diffraction (XRD)

Crystallinity was examined using a Bruker D-8 Advance X-ray diffractometer (Germany) at 40 kV voltage, 40 mA, and copper (Kα) radiation (1.5406 Å). Crystallinity indices were calculated using Eqs. 3 and 4.

3 $$Cr.I.{\text{ }}\left( \% \right){\text{ }} = ~\frac{{Sc}}{{St}} \times {\text{ }}100$$

4 $$\Delta {\text{Cr}}.{\text{I}}.{\text{~}}\left( {\text{\% }} \right){\text{~}} = {\text{~}}\frac{{Cr.l~of~modified~sample - Cr.l~of~unmodified~sample}}{{Cr.l~of~modified~sample}} \times 100$$

The change in crystallinity is represented by ΔCr.I. (%), where St is the total domain area and Sc is the area of the crystalline domain^34,52^. Bragg’s formula was employed to calculate the d-spacing, or distance between crystal planes, while Scherrer’s equation was utilized to calculate the size of the crystallites based on the peak broadening in the diffraction pattern Eqs. 5 and 6:

5 $$\:\text{d}\:\left(\text{n}\text{m}\right)\:=\frac{{\uplambda\:}}{2\:sin\theta\:}$$

6 $$\:\text{C}\text{r}\text{y}\text{s}\text{t}\text{a}\text{l}\:\text{s}\text{i}\text{z}\text{e}\:\left(\text{n}\text{m}\right)\:=\:\frac{0.9\:{\uplambda\:}}{{\upbeta\:}\:Cos\theta\:}$$

where λ = X-ray wavelength (0.154060 nm) and β & and θ represent the full widths at half maxima and Bragg’s angle of the XRD peak, respectively^34,53^.

#### Toxicity evaluation – brine shrimp lethality assay

To hatch A. salina brine shrimp, eggs were incubated in artificial seawater with aeration. The larvae were transferred into containers with sample solutions. To preserve their purity, the A. salina was collected using a Pasteur glass pipette from the illuminated compartment while their shells were left in the darker chamber. In order to make 10 ml of sea water, ten 48-h nauplii were counted and put into bottles. A final volume of 10 ml was created by incubating them in 0, 10, 100, 250, 500, and 1000 mg/L of the components under examination. Tests were carried out in triplicate for every substance concentration and Mortality percentage was calculated using Eq. 7^54^.

7 $${\text{Percentage of mortality }}\left( \% \right):\left( {{\text{Total}}A.{\text{ }}salina{-}{\text{ Alive}}A.{\text{ }}salina} \right){\text{ }} \times {\text{ 1}}00\% /{\text{Total}}A.{\text{ }}salina$$

#### Moisture content and the water solubility of films

Weight loss was measured to estimate the moisture content. Composite films and modified non-woven that had been previously weighed were sliced into 2 cm^2^ squares and baked for 50 min at 106 °C. The final weight of the dry composite films and modified non-woven samples was then recorded. Equation 8 was used to calculate the moisture content:

8 $${\text{Moisture Content in }}\left( \% \right) = x{\text{1}}00$$

Where, Wi = initial weight and Wf = final weight.

The moisture content of the films was found to range between 8.45 and 12.97%. The water solubility of film can be measured as a function in the swelling degree and gel fraction. The swelling degree quantifies the percentage increase in the film weight when left in water, absorbing as much as possible. Conversely, the gel fraction indicates the remaining weight of the swelled film after removing it from the water and subjecting it to a hot medium to evaporate the absorbed water.

#### Contact angle measurement

Contact angle measurements were performed using the GA-1102 Optical Contact Angle instrument, manufactured by Hunan Gonoava Instrument Co. Ltd. (Changsha, China). An average of 250 µL of water was applied to the sample surface under analysis. The test was conducted at a relative humidity of 65 ± 5% and an ambient temperature of 22 ± 1 ºC. The increased measurement uncertainty was ± 1^o^ (coverage factor (k = 2)) at a 95% confidence level^12,55^.

#### Thermogravimetric analysis (TGA/DTG)

The thermal stability of composite film samples was examined using thermogravimetric analysis (TGA). The Shimadzu TGA-50 device was used to determine the weight loss of the composite film samples as a function of temperature. Differential thermogravimetry (DTG), a TGA derivative, was utilized to determine the rate of weight change with temperature. This method is particularly useful for identifying thermal events like decomposition or combustion. Sample masses ranged between 11 and 34 mg. The test was conducted at a flow rate of 30 ml/min, a heating rate of 10 °C/min, and a temperature range of 30 to 1000 degrees Celsius in an inert nitrogen atmosphere^56,57^.

#### Antibacterial activity

The antibacterial activity of the produced films, NWC fabric, and control was assessed using the disc diffusion method (AATCC Test Method 147–2004). Results were expressed in terms of the inhibition zone diameter (mm). *Staphylococcus aureus* ATCC 6538-P Gram-positive, *Escherichia coli* ATCC 25,933 Gram-negative, *Candida albicans* ATCC 10,231 (yeast), and *Aspergillus niger* NRRL-A326 (fungus) were the four representative test microorganisms employed. For yeast and bacteria, 0.1 ml of 10^5^–10^6^ cells/ml was used to inoculate nutrient agar plates evenly. To assess the antifungal activity, 0.1 ml (10^6^ cells/ml) of the fungal inoculum was planted into Czapek-Dox agar plates. In modified weave samples the inoculation plates were covered with treated discs (15 mm in diameter). The inoculation plates were covered with 15 mm-diameter textile treated discs. After that, plates were stored for two to 4 h at a low temperature (4 °C) to maximize diffusion. After that, the plates were incubated for 24 h at 37 °C for bacteria and yeast and 48 h at 30 °C for fungi while upright to promote maximal growth of the organisms. By measuring the diameter of the zone of inhibition and expressing the result in millimeters (mm), the antibacterial activity of the test agent was ascertained. Each experiment was performed multiple times^12,56^, and the average measurement was recorded.

## Results and discussion

### Preparation and characterization of the PVA/SC film

#### Factors affecting the formation of the PVA/SC film

##### PVA/SC weight ratio

**Fig. 1:**
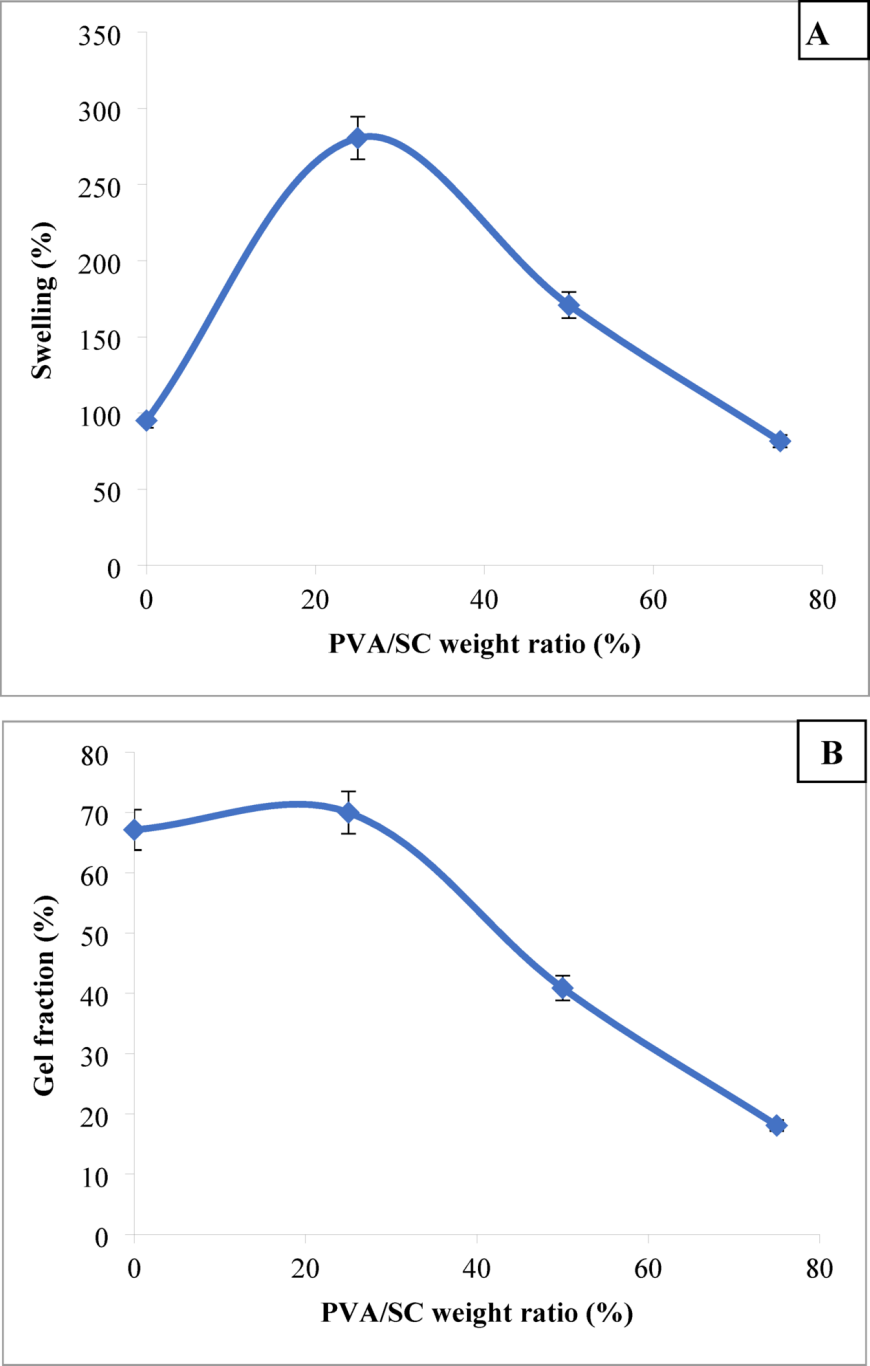
Effect of PVA/SC weight ratio on percent swelling (**A**) and gel fraction (**B**) of the PVA/SC film, [SC], 6%; [PVA], 6%; [acetic acid], 0.5%; immersion time acetic acid, 10 min in.

The effect of the PVA/SC weight ratio on percent swelling and gel fraction of the PVA/SC films is illustrated in Fig. 1A,B respectively. The addition of 25% PVA to SC (w/w) increased the percent swelling and gel fraction of the films to 281% and 70%, respectively. This effect appears to stem from the formation of secondary bonds between the PVA macromolecules and casein macromolecules. These bonds enhance molecular chain entanglements and hydrogen-bonding networks within the film structure^58^. However, as the PVA ratio increased from 25 to 75%, both the swelling properties and gel fraction showed a reduction. This suggests a gradual dissociation of the film structure, which may be attributed to a decrease in chain entanglements among the casein macromolecules. Consequently, the reduction in intermolecular bonds weakens the overall network within the film^20^.

##### Acetic acid concentration

**Fig. 2:**
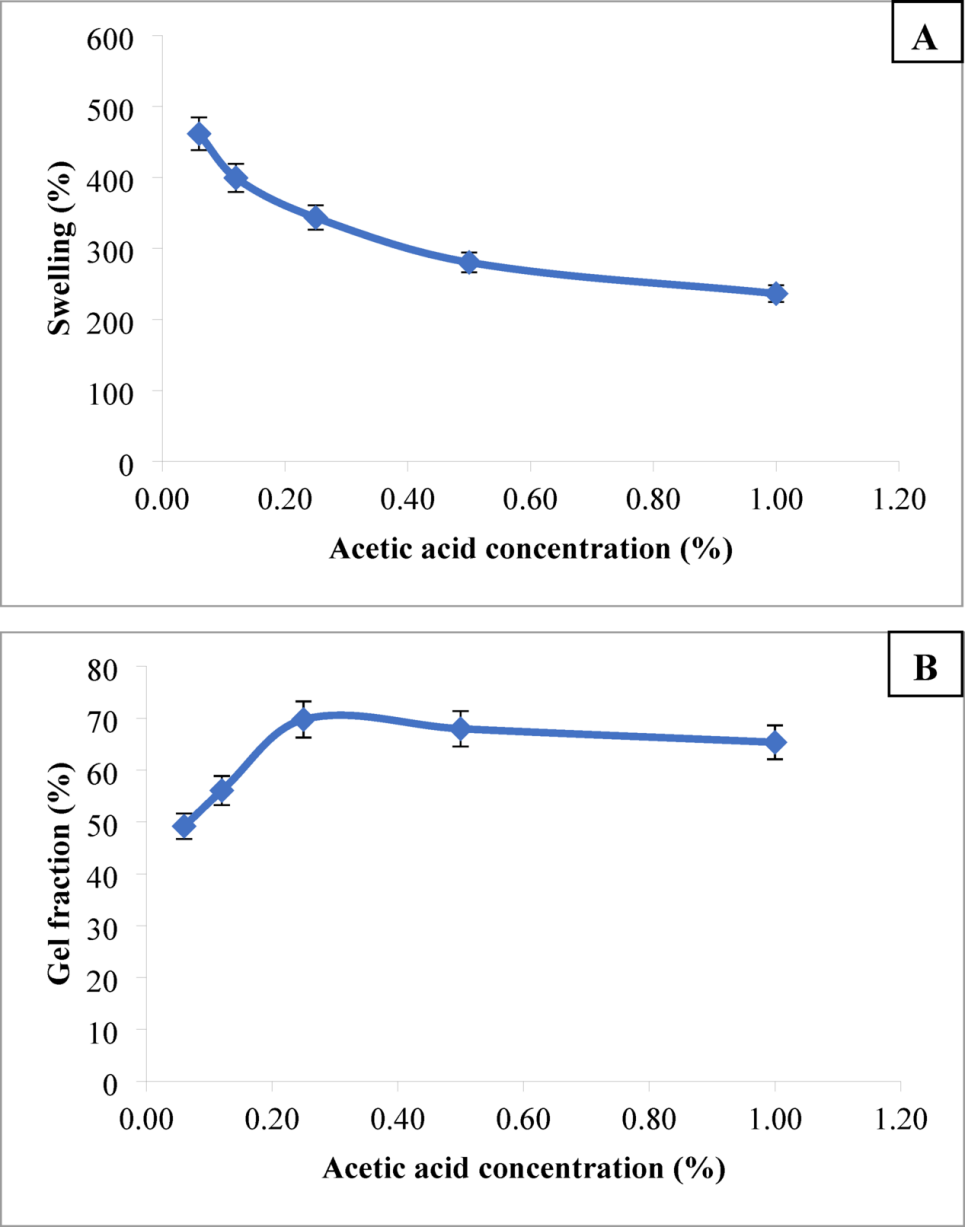
Effect of acetic acid concentration on percent swelling (**A**) and gel fraction (**B**) of the PVA/SC film, [SC], 6%; [PVA], 6%; PVA/SC weight ratio, 25/75; immersion time in acetic acid, 10 min.

The impact of soaking of PVA/SC (25/75, respectively) film in varying concentrations of acetic acid on their percent swelling and gel fraction of that film is illustrated in Fig. 2A, B, respectively. It is evident that increasing the acetic acid concentration from 0.06 to 0.25% (v/v) leads to a gradual decrease in percent swelling accompanied by an increasing in gel fraction of the film. Beyond the acetic acid concentration of 0.25, within the studied range, both properties show a noticeable decline. The increase in acetic acid concentration to 0.25% neutralizes the casein sodium salt, causing its precipitation and consequently altering the film’s swelling and gel fraction properties^23,59^. Beyond 0.25%, the PVP/SC film gradually dissolves, leading to the reduction in the extent of these properties in the treated film.

#####  Immersion time

**Fig. 3:**
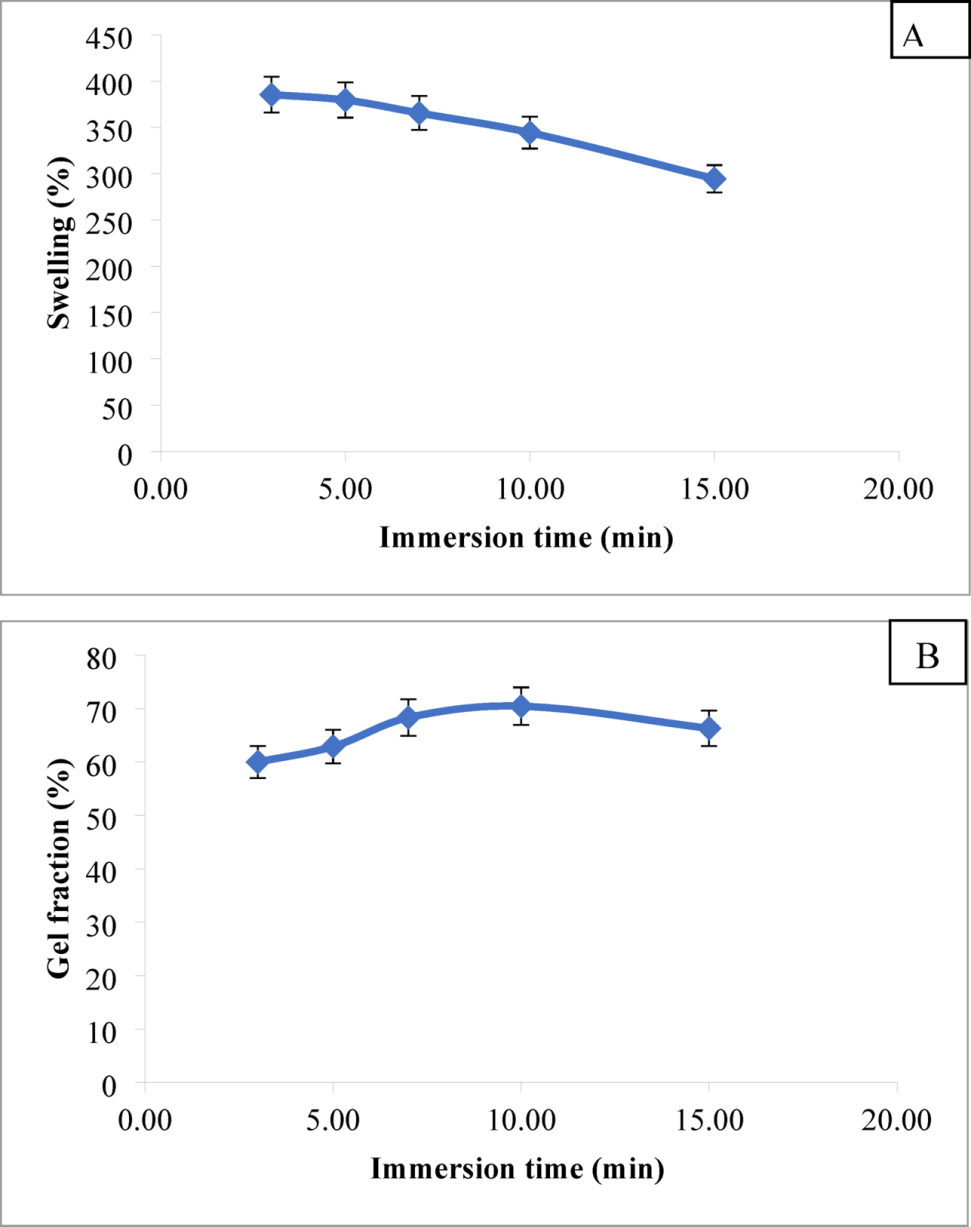
Effect of immersion time in acetic acid on percent swelling (**A**) and gel fraction (**B**) of the PVA/SC film, [SC], 6%; [PVA], 6%; PVA/SC weight ratio, 25/75; [acetic acid], 0.25%.

The impact of immersion time of the PVP/SC film in 0.25% acetic acid on percent swelling and gel fraction of such film is illustrated by Fig. 3A and B, respectively. It is evident that increasing the immersion time from 3 to 10 min leads to an increase in gel fraction, accompanied by a reduction in percent swelling of the film. This suggests an enhancement in hydrogen bonding and protein-protein interaction within the film structure over time, resulting in greater firmness^45,60^. However, beyond an immersion time of 10 min, both properties show a decline, indicating the gradual solubility of the film under these conditions.

#### Addition of curcumin, shellac, silver nanoparticles or their admixtures as bio-additives to the PVA/SC films

**Fig. 4:**
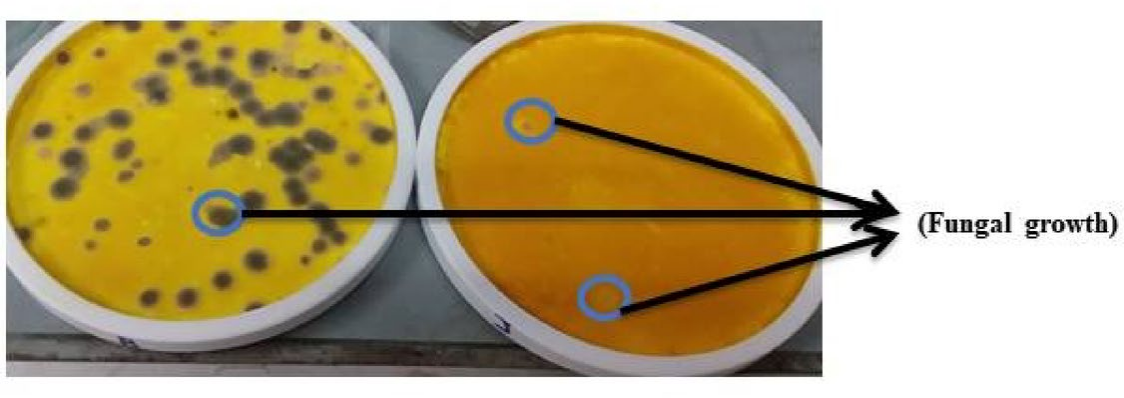
The fungal growth on surfaces of PVA/SC films loaded with 0.05 and 0.075% of curcumin and dried at room temperature.

**Fig. 5:**
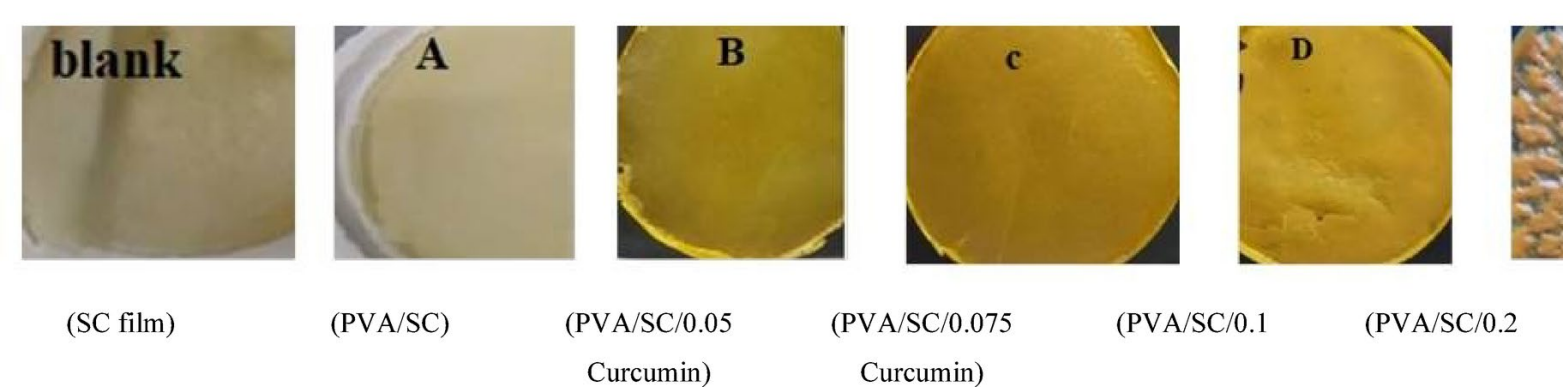
The visual appearance of PVA/SC films loaded with different concentrations of curcumin.

During practical observations, fungal growth was noticed on surfaces of PVA/SC films loaded with curcumin when drying at room temperature (Fig. 4). To address this issue, it was necessary to dry such films in an oven at 45 ^o^C. Figure 5 shows that increasing the curcumin concentration from 0 to 0.075% in the PVA/SC film formulation results in successful film formation (Fig. 5B and C). However, as the curcumin concentration increases to 0.1%, the film begins to show signs of poor quality (Fig. 5D). At a higher curcumin concentration of 0.2%, the films become cracked and unusable (Fig. 5E). The effect of incorporating curcumin, shellac, silver nanoparticles or their combinations as bio-additives in the PVA/SC film formulation on percent swelling and gel fraction is summarized in Table 2. Increasing the curcumin concentration from 0.05 to 0.075% in the PVA/SC film formulation, i.e. D2 and D3, reduces the swelling and gel fraction. This reduction is attributed to the hydrophobic nature and molecular size of curcumin, which disrupts the intermolecular hydrogen and hydrophobic bonds within the PVA/SC film structure, leading to gradual solubilization of the film^45^.

When the curcumin concentration is increased to 0.1% (D4), both the percent swelling and gel fraction values drop to zero, suggesting the complete solubilization of the film. Moreover, the inclusion of shellac as a hydrophobic bio-additive to the PVA/SC film formulation (D5) results in decreased swellability and gel fraction. This is likely due to partial disruption of the H-bonds and the protein-protein interactions within the film structure^45,60^. Furthermore, combining of curcumin with shellac in the PVA/SC film structure (D7 and D8) enhances the film’s gel fraction, indicating increased firmness, while marginally reducing the swelling degree. This is in contrast to the film formulations of D2 and D3 containing only the curcumin as a bio-additive, suggesting a synergistic effect of combining curcumin and shellac in the PVA/SC film structure. Conversely, the inclusion of the Ag-NPs, regardless of its concentration, in any of the above PVA/SC film formulations, (D6 and D9-12) disrupts the hydrogen bonds within the film structures, thereby reducing their stability and cohesion.

**Table 2:** Effect of the bio-additive type and concentrations on percent swelling and gel fraction of the PVA/SC film.

Designation of Film formulation	Curcumin (%)	Shellac (%)	Ag-NPs (%)	SW%	GF
D1	0	0	0	271.1 ± 3.12	70.4 ± 1.25
D2	0.05	0	0	145.2 ± 2.24	54.3 ± 1.22
D3	0.075	0	0	113.4 ± 1.51	51.9 ± 1.34
D4	0.100	0	0	0	0
D5	0	10.0	0	35.27 ± 2.22	47.16 ± 2.43
D6	0	0	3.0	0	0
D7	0.075	7.5	0	141.19 ± 1.36	64.2 ± 2.13
D8	0.075	15.0	0	136.81 ± 1.24	70.1 ± 1.43
D9	0.075	0	3.0	91.86 ± 2.11	39.73 ± 1.74
D10	0.075	0	6.0	153.46 ± 1.31	54.45 ± 1.11
D11	0	10.0	1.5	75.19 ± 1.11	62.57 ± 1.24
D12	0	10.0	2.5	67.99 ± 1.37	54.08 ± 2.41
LSD at 0.05%				10.23	6.71

[SC], 6%; [PVA], 6%; PVA/SC weight ratio, 25/75; [acetic acid], 0.25%; at 10 min.

#### Characterization of the PVA/SC film using FTIR analysis

**Fig. 6:**
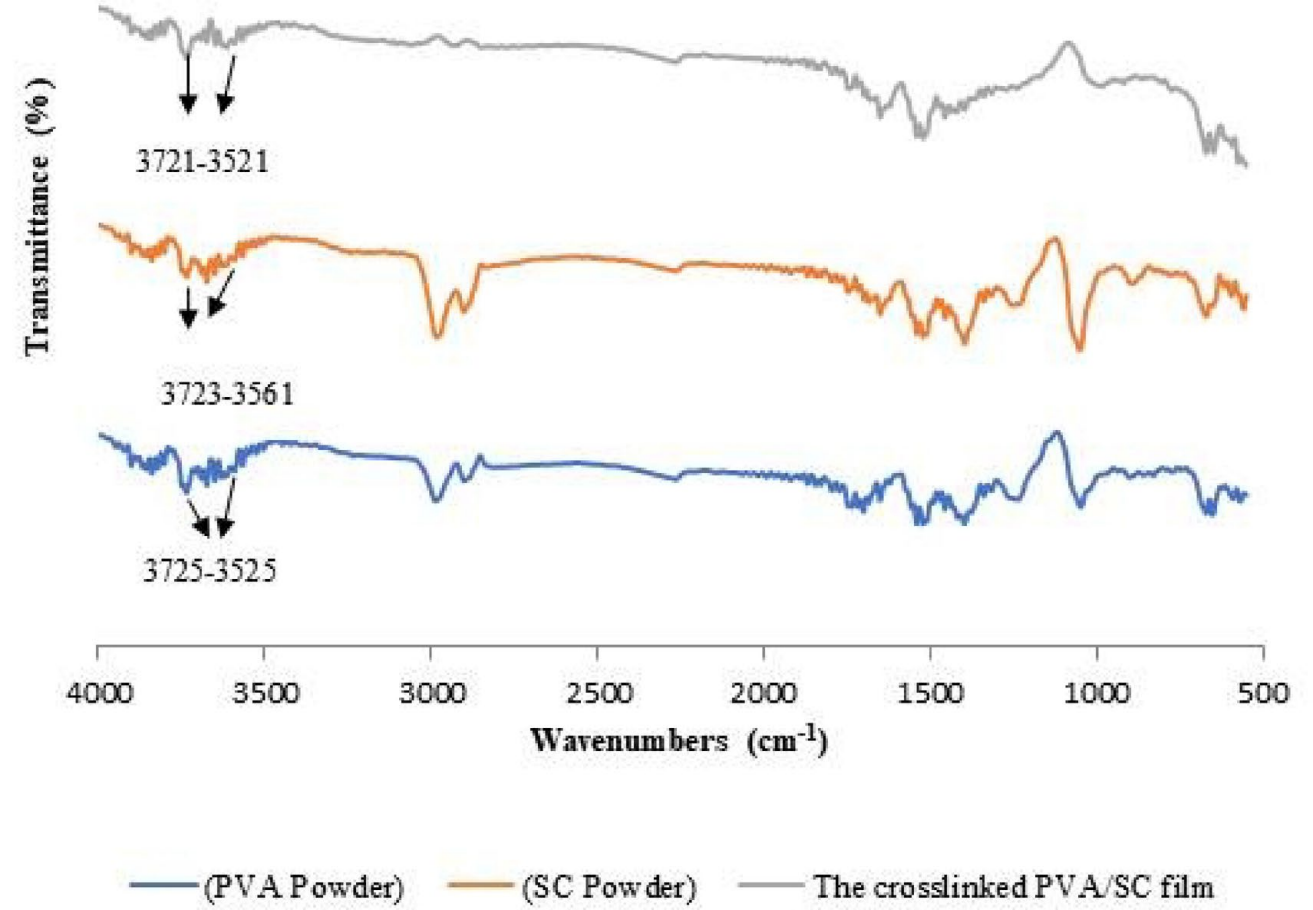
FTIR analysis of PVA, SC and the crosslinked PVA/SC film.

Figure 6 shows the FTIR analysis of PVA, sodium caseinate, and the crosslinked PVA/SC film. The PVA spectrum reveals a peak at 3736 cm^− 1^, corresponding to the stretching vibration of OH groups, and another peak at 2981 cm^− 1^, representing the asymmetric stretching vibration of CH_2_ groups. Moreover, it includes peaks at 1702 cm^− 1^ corresponding to the stretching vibration of C = O groups, and at 1045 cm^− 1^ C-O for stretching of the acetyl groups as the PVA used (Mowiol 4–80) is partially hydrolyzed. The FTIR of the sodium caseinate spectrum includes peaks in the range of 3725 –3561 cm^− 1^, corresponding to the amino groups^61^. It also exhibits peaks at 2980 cm^− 1^, corresponding to CH_2_ groups, along with distinctive peaks of amide I at 1630 cm^− 1^ and amide II at 1529 cm^− 1^^61^. Furthermore, the crosslinked PVA/SC film spectrum combines characteristic peaks from both PVA and SC. It includes a peak at 3644 cm^− 1^, corresponding to the amino groups of SC, a peak at 2980 cm^− 1^ for CH_2_ groups of PVA, peaks of the amide I at 1630 cm^− 1^ and amide II at 1525 cm^− 1^. These features confirm the successful formation of the crosslinked PVA/SC film.

### Treating of the non-woven cotton fabric with PVA/SC/bio-additives formulations

####  Performance properties of the treated non-woven cotton fabric

Table 3 shows the impact of treating non-woven samples with the PVA/SC (25/75, respectively) blend loaded with curcumin, shellac, Ag-NPs or their combinations on the performance properties of the treated fabric. Beyond enhancing air permeability by 56.4, 82.2, and 63.2%, the findings of tensile strength and Young’s modulus increased by 1.21, 140% respectively following the addition of PVA/SC. Furthermore, increasing the curcumin concentrations from 0.05 to 0.075% led to notable improvements in TS (by 74% and 190%) and YM (by 205% and 488%, respectively) compared to untreated non-woven fabric. Additionally, the burst strength increased by 82% after adding PVA/SC and further increased by 160, and 114% with higher curcumin concentrations, confirming the previously mentioned results. The film’s percent swelling and gel fraction values reduced to zero at curcumin concentrations above 0.075 and up to 0.1%, indicating complete film solubilization. It is clear that: (a) increasing of curcumin concentration from 0 to 0.075% in the PVA/SC formulation resulted in improved swellability, gel fraction, tensile strength, Young’s modulus, and air permeability, alongside reduced burst strength and wettability of the treated fabric. This can be attributed to the formation of the enol form of curcumin (Schema 1) during treatment with aqueous acetic acid prior to drying. This process enhances intermolecular hydrogen bonds within the PVA/SC/CUR film structure, resulting in a slight increase in the gel fraction compared to the PVA/SC film^62^, (b) increasing of Ag-NPs from 3 to 6% in the above formulation containing 0.075% of curcumin results in an increase in swellability, gel fraction, burst along with a decrease in tensile strength, Young’s modulus, air permeability, and wettability of treated fabric. This behavior can be attributed to the hydrophobic characteristics of curcumin in addition to its molecular size that breaks down the H-bonds within the PVA/SC blend structure, leading to a gradual solubilization of the PVA/SC film as previously mentioned^63^, (c) increasing of Ag-NPs from 0 to 1.5% in the PVA/SC formulation containing 10% enhances the gel fraction, tensile strength, Young’s modulus; while decreasing swellability, air permeability, burst, and wettability. This effect can be explained by the formation of a firm coating layer on the treated fabric, composed of the formulation ingredients. (d) increasing of Ag-NPs from 1.5 to 2.5% in the PVA/SC formulation in the above mentioned formulation results in higher tensile strength, air permeability, and burst, while simultaneously reducing swellability, gel fraction, Young’s modulus, and wettability of treated fabric suggesting a partial solubilization of the coating layer as a result to increasing of the Ag-NPs concentration, (e) increasing of shellac concentration from 10 to 15% in the PVA/SC formulation containing 0.075% of curcumin leads to enhanced swellability, tensile strength, burst, along with reduced gel fraction, Young’s modulus, air permeability, and wettability. 

**Schema 1:**
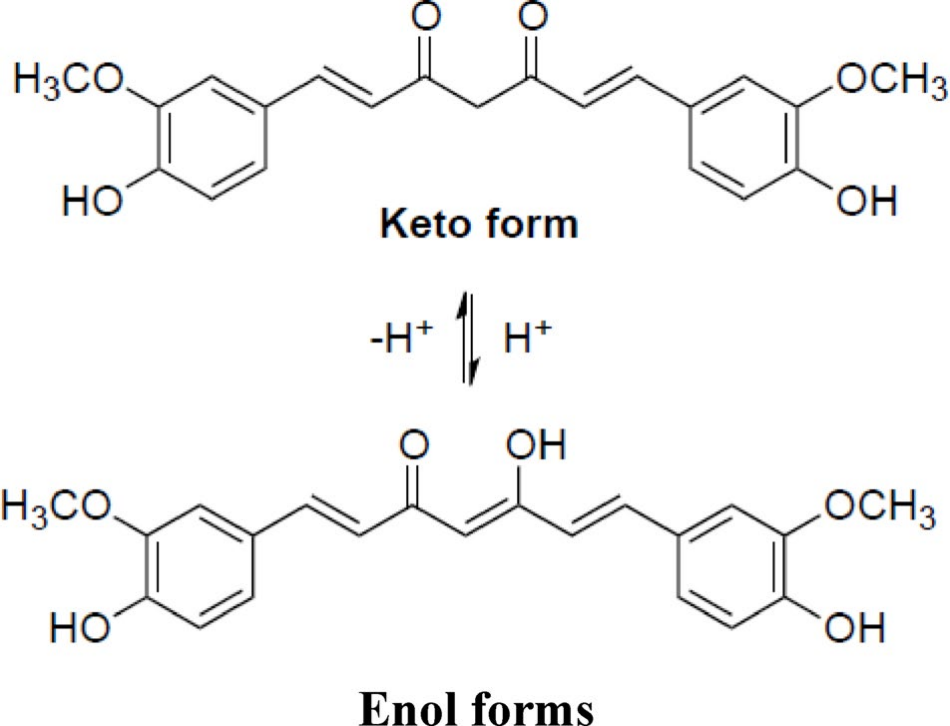
The chemical structure of keto and enol forms of curcumin.

This reflects the formation of a tight coating layer on the fabric surface, attributed to the hydrophobic nature and molecular structure of shellac, which reinforces hydrogen bonds within the PVA/SC film^33–36^, and f) the non-woven cotton fabric coated with PVA/SC/CUR(0.075)/SH(10%) formulation exhibits the best gel fraction performance. This can be attributed to the heating effect on the NWC/PVA/SC/CUR(0.075)/SH(10%) film for the drying purpose that may cause shellac to undergo self-estrification due to the interaction among its hydoxyl and carboxyl groups forming polymerized shellac^64^. This creates a network structure in the shellac, which, together with increased H-bonds from the curcumin enol, enhances the gel fraction of the NWC/PVA/SC/CUR/SH film.

**Table 3:** Effect of curcumin/shellac/Ag-NPs concentrations on percent swelling gel fraction and mechanical properties of modified non-woven fabric.

Designation of film formulation	Curcumin (%)	Ag-NPs (%)	SW (%)	GF	Tensile strength (MPa)	Young’s Modulus (MPa)	Air permeability (cm^3^/ cm^2^.S)	Burst (kg/cm^2^)
NW0	0	0	247.30	92.70	13.17	31.77	231.80	1.097
NW1	0	0	239.00	66.44	13.33	76.52	101.00	2.000
NW2	0.05	0	171.90	66.50	22.87	96.91	41.20	2.850
NW3	0.075	0	221.80	68.43	38.20	187.0	85.05	2.347
NW4	0.075	3.0	250.20	72.41	36.15	69.83	75.30	1.403
NW5	0.075	6.0	276.60	77.23	15.82	21.87	65.50	1.600
	Shellac (%)	Ag-NPs (%)						
NW6	10	0	189.50	71.52	15.64	18.38	83.30	1.647
NW7	10	1.5	179.20	82.56	33.03	64.97	53.00	1.543
NW8	10	2.5	156.00	66.67	38.70	62.42	65.10	1.743
	Curcumin (%)	Shellac (%)						
NW9	0.075	10.0	190.20	95.79	35.61	67.78	48.00	2.133
NW10	0.075	15.0	202.50	75.34	39.37	67.35	47.20	2.220
LSD at 0.05%			21.09	7.54	3.12	6.89	9.13	0.26

**Fig. 7:**
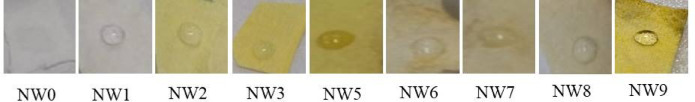
Wettability by water droplet after crosslinking of treated non-woven with 0.25% acetic acid.

Table 4; Fig. 7 show visually the wettability of all the aforementioned treated samples. Similar results were obtained using the layer-by-layer assembly technique, which was employed to fabricate novel non-woven cotton fabric with multiple layers of hyaluronic acid (HA) and chitosan. In this method, the air permeability and relative water vapor permeability of treated fabric decreased, whereas, the gel fractions, antibacterial activities, and swelling properties increased with an increase in HA concentration from 0.25 to 1.0% ^20^. Moreover, Aloe Vera was encapsulated with corn starch using the simple coacervation technique and incorporated into non-woven cotton fabric by using butane tetra-carboxylic acid as binding agent to enhance the antibacterial activities, cosmetic effects, UV protection, and medication application^65^. Generally, the optimal matrix in terms of gel fraction is the non-woven cotton fabric coated with PVA/SC/CUR(0.075%)/SH(10%) formulation.

**Table 4:** Effect of curcumin/shellac/Ag-NPs concentrations on percent wettability properties of modified non-woven fabric.

Designation of film formulation	Curcumin (%)	Ag-NPs (%)	Wettability before AC (S)	Wettability after AC (S)
NW0	0	0	0.5 ± 0.27	2 ± 0.45
NW1	0	0	27 ± 0.44	600 ± 2.38
NW2	0.05	0	36 ± 1.21	1200 ± 3.16
NW3	0.075	0	23 ± 2.25	2100 ± 0.51
NW4	0.075	3.0	6 ± 0.56	1500 ± 1.33
NW5	0.075	6.0	6 ± 0.23	4320 ± 1.61
	Shellac (%)	Ag-NPs (%)		
NW6	10	0	3 ± 0.21	4860 ± 3.22
NW7	10	1.5	11 ± 1.87	4920 ± 3.21
NW8	10	2.5	5 ± 0.56	7380 ± 3.5
	Curcumin (%)	Shellac (%)		
NW9	0.075	10.0	5 ± 0.31	2100 ± 2.64
NW10	0.075	15.0	8 ± 0.11	2760 ± 2.35
LSD at 0.05%			1.19	243.52

[SC], 6%; [PVA], 6%; PVA/SC weight ratio, 25/75; [acetic acid], 0.25%; immersion time, 10 min.* NW0* untreated non-woven cotton fabric,* NW1* non-woven cotton fabric treated with PVA/SC blend in the absence of any of the bio-additives,* AC* acetic acid.

This superior performance can be attributed to the heating effect applied to the NWC/PVA/SC/CUR(0.075%)/SH(10%) film during the drying process. The heating may induce self-esterification of shellac, caused by interactions between its hydroxyl and carboxyl groups, resulting in the formation of polymerized shellac. This polymerized shellac creates a network structure, which, when combined with enhanced hydrogen bonding due to the curcumin enol form, significantly improves the gel fraction of the NWC/PVA/SC/CUR/SH film.

#### Characterization of the treated non-woven cotton fabric

#####  FTIR analysis of the treated non-woven cotton fabric

**Fig. 8:**
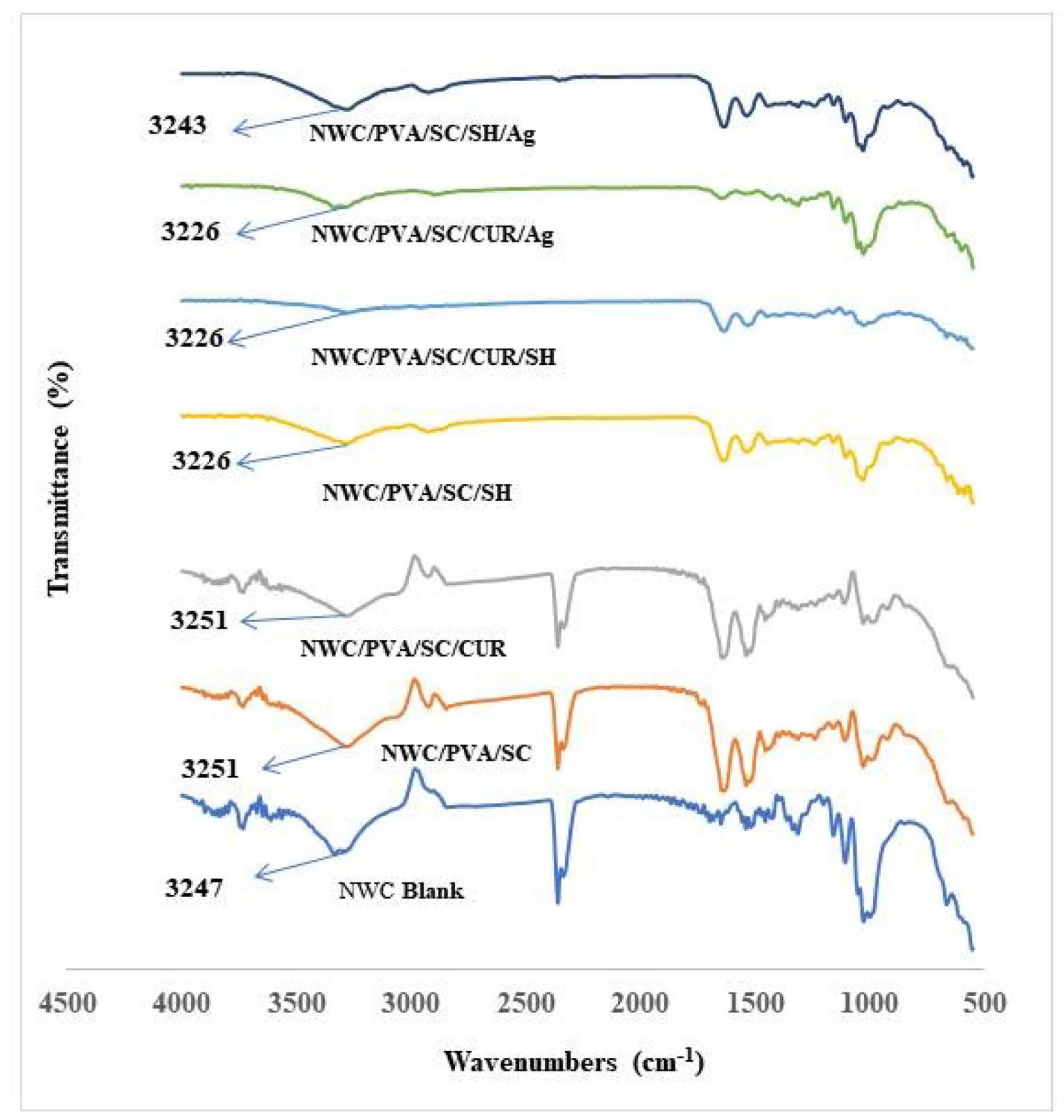
The FTIR analysis of the non-woven treated with PVA/SC formulations containing the curcumin, shellac, Ag-NPs and/or their combinations.

Figure 8 illustrates the FTIR analysis of the non-woven cotton fabric treated with PVA/SC formulations containing curcumin, shellac, Ag-NPs, and their combinations. The FTIR spectrum of the untreated blank non-woven cotton fabric spectrum includes a peak at 3247 cm^− 1^, corresponding to the stretching vibration of the -OH group, and a peak at 1025 cm^− 1^, representing the C–O–C stretching vibration of the glucose unit^47^.

For the non-woven cotton fabric treated with the PVA/CS blend, the -OH stretching vibration is observed at 3251 cm^− 1^. However, the incorporation of shellac and/or curcumin into the PVA/CS blend alters the intensity of this peak. Moreover, the FT-IR spectra of the NWC/PVA/SC blend includes two peaks at 1538 and 1633 cm^− 1^, which are attributed to the anti-symmetric stretching and stretching vibrations of the COOH group^66^. These peaks are absent in the untreated NWC fabric spectrum, confirming the treatment of the fabric with the PVA/SC blend. The inclusion of shellac or curcumin into the PVA/SC matrix causes variations in the H-bonds within the matrix, resulting in shifts in the wavelengths of functional groups in the formulation^39^. In addition, the binding of curcumin or shellac to the casein is possible through the amide and -OH groups, leading to a decrease in the intensity of these vibrations^67,68^.

Interestingly, peaks at 2500 cm^− 1^ appear in the FTIR spectra of the NWC blank, NWC/PVA/SC, and NWC/PVA/SC films. However, these peaks disappear completely in the spectra of NWC/PVA/SC/SH, NWC/PVA/SC/CUR/SH, NWC/PVA/SC/CUR/Ag and NWC/PVA/SC/SH/Ag. This discrepancy is attributed to an instrumental error in the FTIR analysis.

##### X-ray analysis (XRD)

**Fig. 9:**
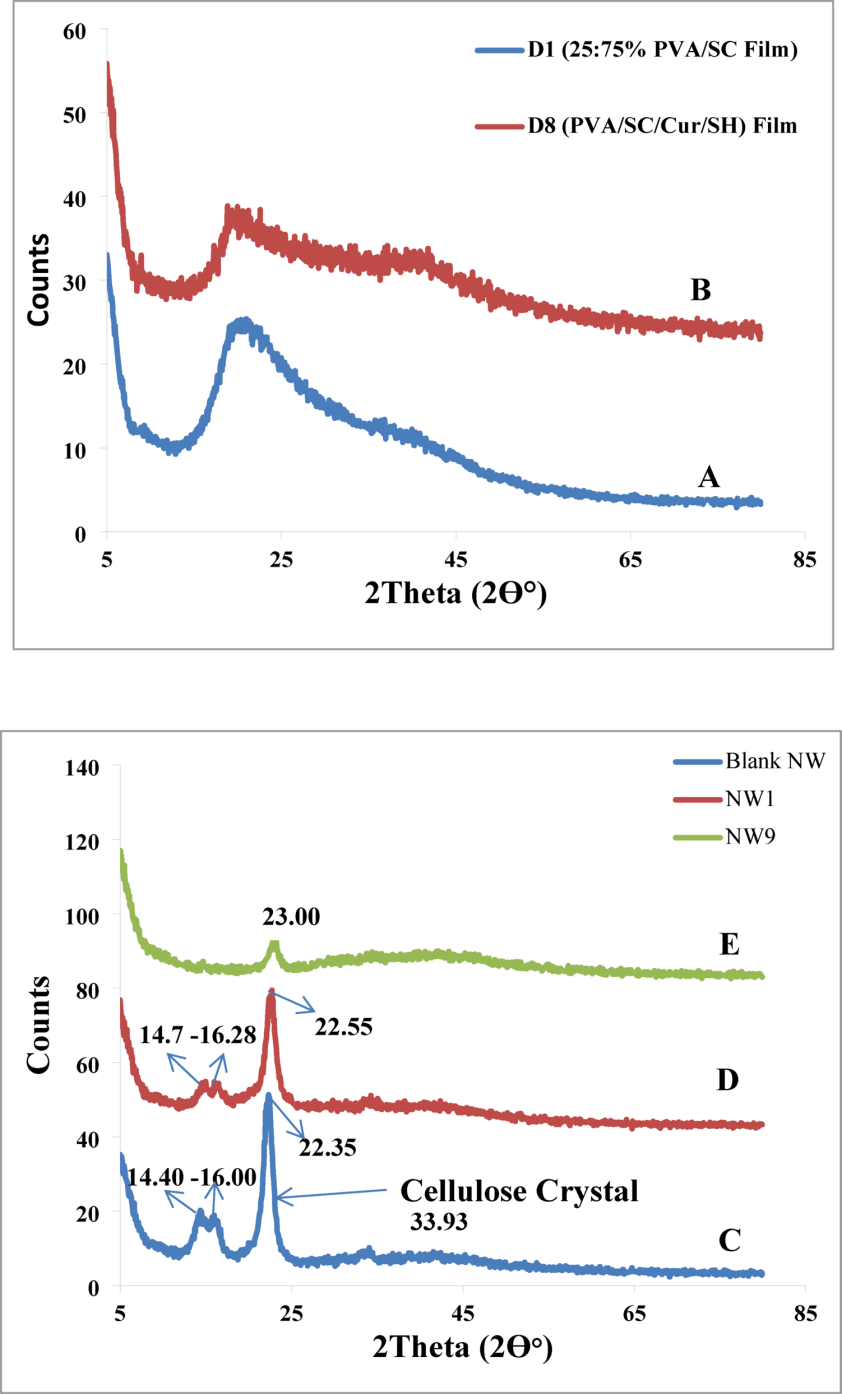
XRD analysis of (**A**) 25:75% PVA/SC Film, (**B**) D8 (PVA/SC/Cur/SH) Film, (**C**) Blank NW Cellulose, (**D**) NW1 and (**E**) NW9.

X-ray diffraction (XRD) is widely used in industrial settings to evaluate materials and samples, atomic-level structures and elements. With a wavelength range of 0.01 to 10 nm, X-rays are ideal for studying atomic-level and elements. Figure 9; Table 5 show the XRD analysis for (A) 25:75% PVA/SC film (D1), (B) PVA/SC/CUR/SH) film (D10), (C) Blank non-woven cellulose, (D) NW1 and (E) NW9. The ratio of the sum of the surface areas under the crystalline peaks to the entire area under the scattering curve was used to calculate the degree of crystallinity (Cr.I, %). The Cr.I (%) values for films D1 (PVA/SC) and D8 (PVA/SC/0.075:15% of CUR: SH) were 3.92 and 5.20%, respectively. This increase is attributed to the additional hydrogen bonds (H-bonds) formed during the crosslinking reaction between PVA/SC, curcumin (CUR), and shellac (SH), leading to enhanced crystallinity. The resulting increase in crystallinity caused a decrease in the overall area of the amorphous region.

For non-woven cellulose, the Cr.I (%) values were 80.3, 78.0, and 12.2% for NW0 (Blank), NW1 (NW0 treated by PVA/SC), and NW9 (NW/PVA/SC/0.075:10% of CUR: SH) respectively^18,34,56^. These results show a significant reduction in Cr.I (%) from 80.3 to 12.2%, confirming the interaction between the PVA/SC/CUR/SH matrix and the non-woven cellulose surface.

The crystallinity of the films alone (Fig. 9A and B) showed low percentages of crystallinity. In contrast, the crystallinity of the non-woven samples before and after treatment with PVA/SC or PVA/SC/0.075:10% of CUR: SH (Fig. 9C–E; Table 5) decreased significantly following treatment. This notable reduction in crystallinity indicates an increase in the amorphous region, validating the successful integration of PVA/SC or PVA/SC/CUR/SH with the non-woven cellulose surface.

For films, it is apparent that the d (nm) values of D8 (PVA/SC/0.075:15% of CUR: SH) increased in comparison to D1 (PVA/SC). While the d (nm) values of NW1 (NW0 treated by PVA/SC) and NW9 (NW/PVA/SC/0.075:10% of CUR: SH) decreased relative to NW0 (Blank) as shown in Table 5. This reduction may be due to the strong crosslinking interaction between the OH and COO- groups of PVA/SC/CUR-SH with OH groups on the non-woven cellulose surface. The resulting strong adhesion forces are more pronounced in the treated non-woven samples compared to the standalone films (Schema 2)^18,56^.

**Table 5:** Crystallinity of films and modified non-woven samples.

	Sample	Cr.l (%)	$\:\varDelta\:$ Cr.l(%)	d (nm)	Crystal size (nm)
Films	D1	3.92	-	0.447	84.20
	D8	5.20	24.62	0.45	9.54
Non-woven	Blank (NW0)	80.3	-	0.54	83.93
	NW1	78.0	− 2.95	0.51	12.19
	NW9	12.2	-558.19	0.39	12.6

**Schema 2:**
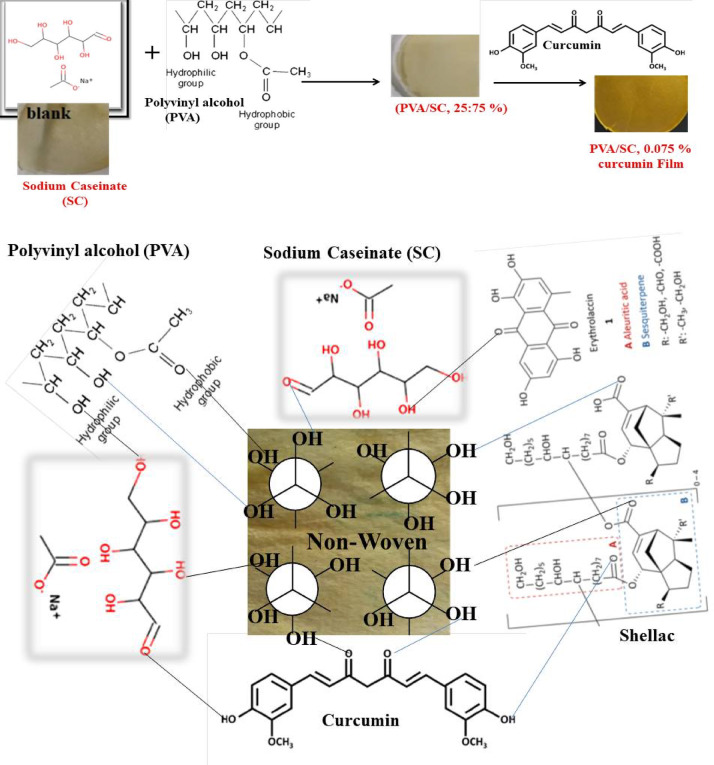

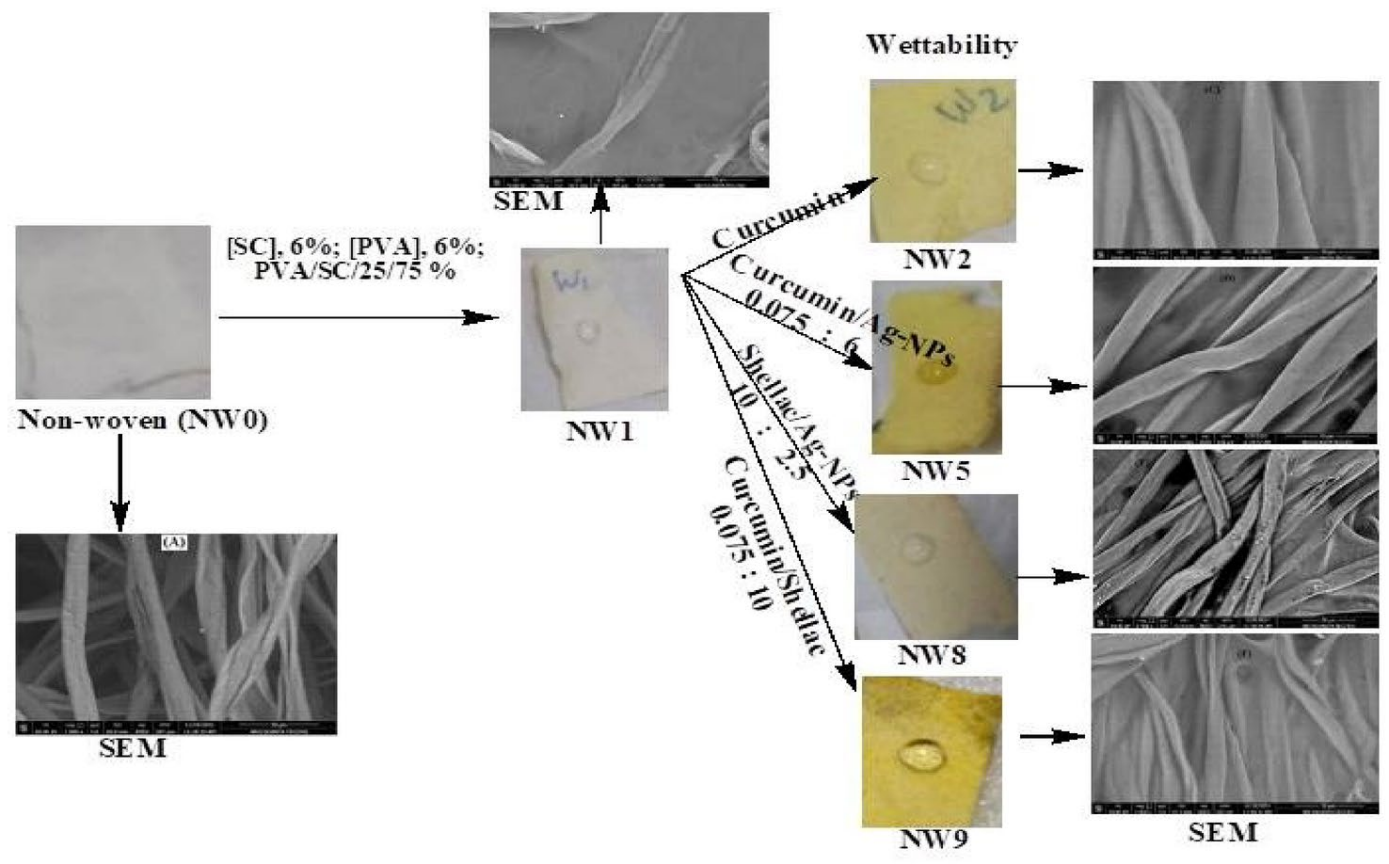
Diagram of the PVA/SC film and treated non-woven fabric formation and there suggested mechanism.

##### SEM and EDX images

**Fig. 10:**
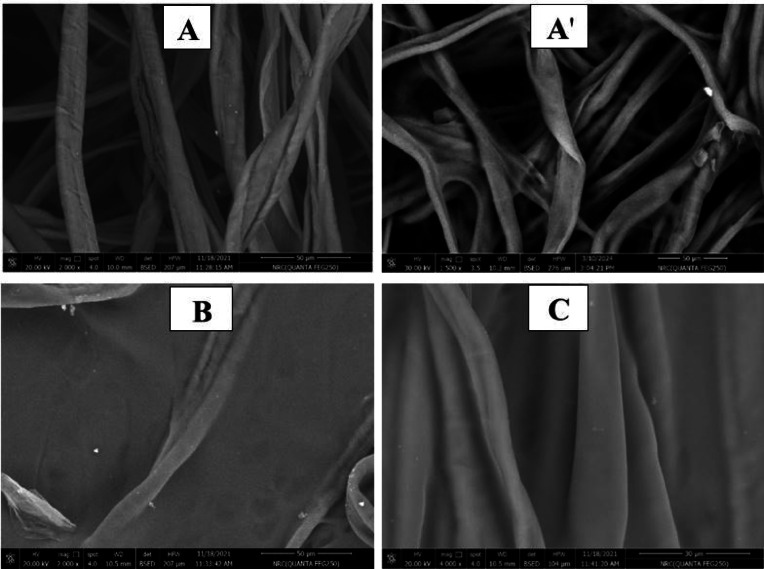

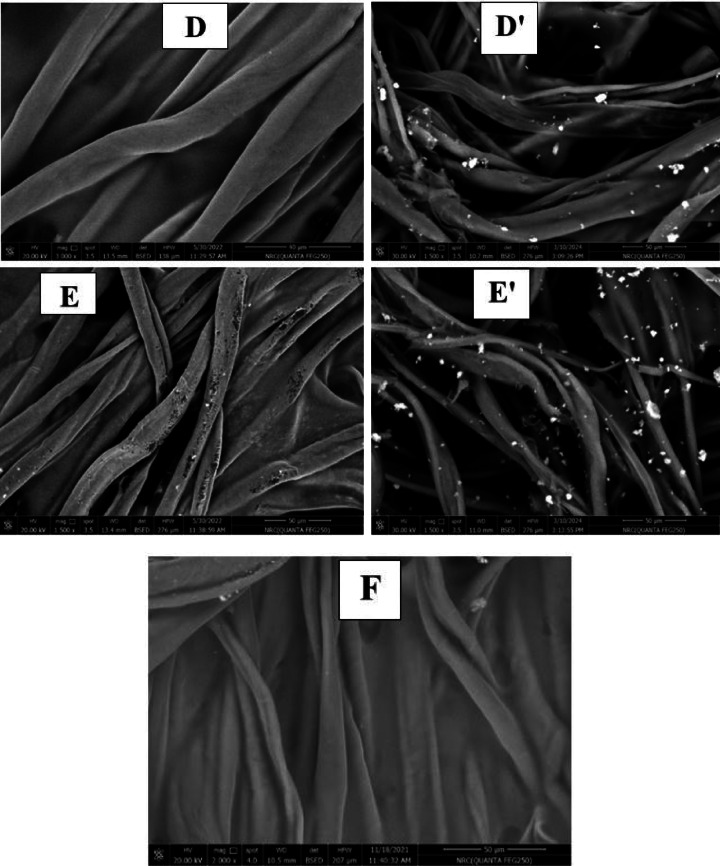
SEM images of (**A** and** A’**) untreated non-woven cotton fabric before and after steeping in H_2_O respectively, (**B**) non-woven cotton fabric treated with PVA/SC formulation, (**C**) non-woven fabric treated with PVA/SC/curcumin formulation, (**D** and** D’**) non-woven fabric treated with PVA/SC/curcumin/Ag-NPs formulation before and after steeping in H_2_O respectively, (**E** and** E’**) non-woven fabric treated with PVA/SC/shellac/Ag-NPs formulation before and after steeping in H_2_O respectively, and (**F**) non-woven fabric treated with PVA/SC/curcumin/shellac formulation.

Figure 10A-F shows the scanning electron microscopy (SEM) images of untreated non-woven cotton fabric and samples treated with PVA/SC formulations containing curcumin, shellac, Ag-NPs and/or their combinations. The SEM images reveal that all the treated fabric surfaces are coated with a layer of PVA/SC formulation ingredients loaded with any of the bio-additives. Moreover, Fig. 10A’, D’ and E’ shows the SEM images of treated samples A, D, and E after being steeped in distilled water for 24 h. Steeping the untreated fabric sample in distilled water results in swelling, while samples D and E exhibit partial dissolution of their coating layers, making the Ag-NPs on the surfaces of these samples more visible.

**Fig. 11:**
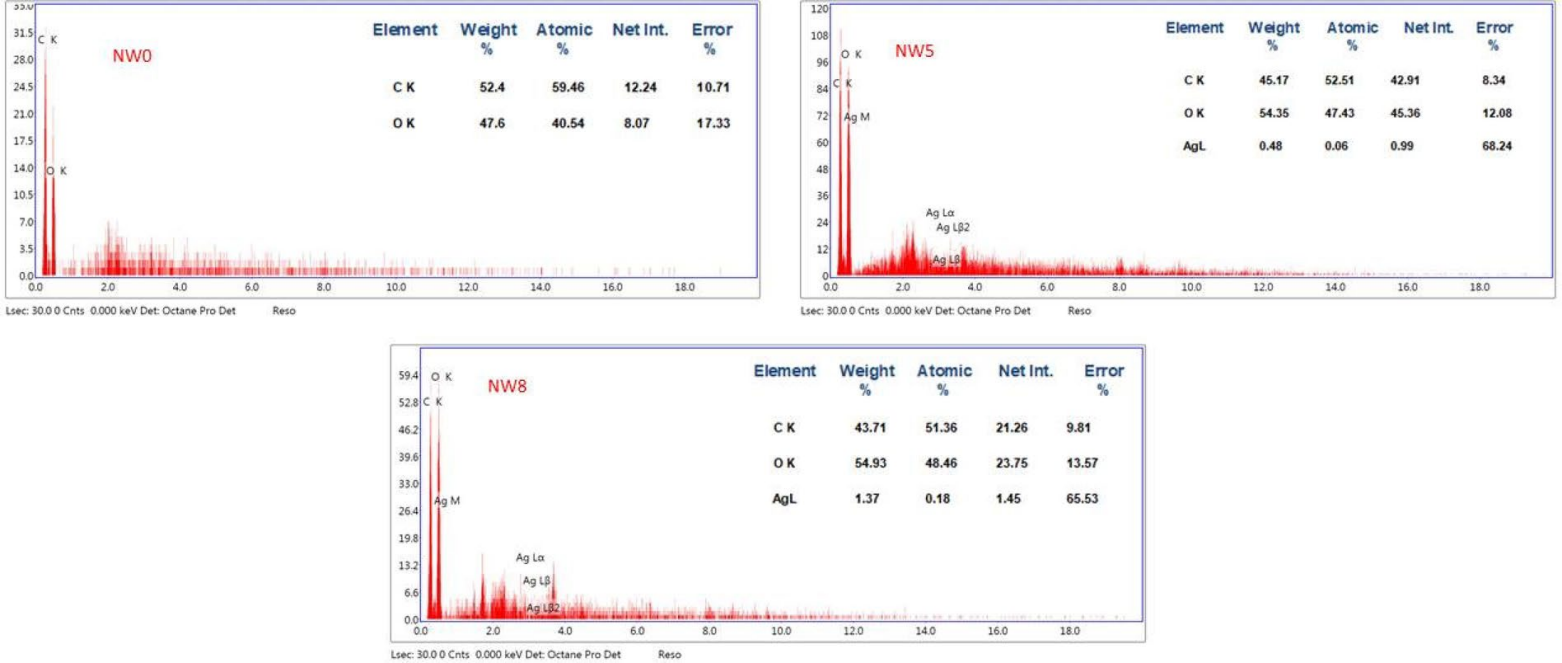
EDX images of untreated non-woven fabrics (NW0), PVA/SC/curcumin/Ag-NPs formulation treated fabric (NW5), and PVA/SC/shellac/Ag-NPs formulation treated fabric (NW8).

On the other hand, Fig. 11 represents the EDX images of untreated non-woven fabric, PVA/SC/curcumin/Ag-NPs treated fabric sample and PVA/SC/shellac/Ag-NPs treated fabric sample. The EDX image of the untreated fabric sample (A) displays only the elements of carbon and oxygen, which are typical of cellulose. However, the EDX images for both treated samples (B and C) show the presence of silver, in addition to carbon and oxygen, confirming the successful incorporation of Ag-NPs into the treated fabric.

##### Thermogravimetric analysis (TGA/DTG)

The samples’ thermal characteristics were analyzed using TGA. Polymers degrade at specific temperatures and times based on their thermal stability, which depends on the interactions between macromolecules and low molecular weight molecules. The interactions between these molecules, such as dipole-dipole, hydrogen, and van der Waals bonds, influence their stability. When thermal energy exceeds bond energy, chain scission and bond dissociations begin^69^. In thermogravimetric analysis (TGA), derivative thermogravimetric curves (DTG), are employed. TGA measures mass change as a function of temperature, whereas DTG reveals the rate of mass loss over temperature, allowing distinction between thermal phenomena like decomposition, oxidation, or desorption.

**Fig. 12:**
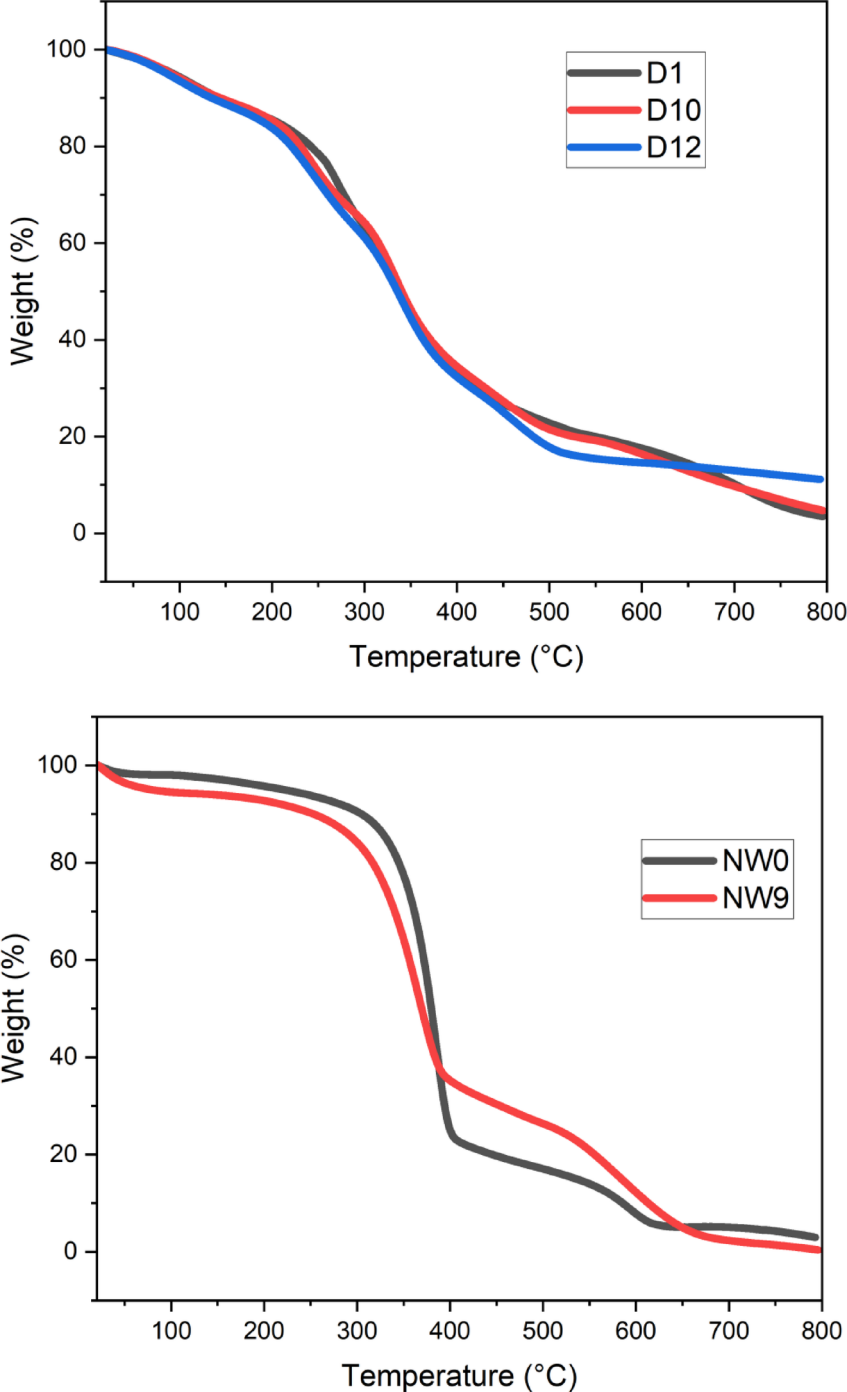

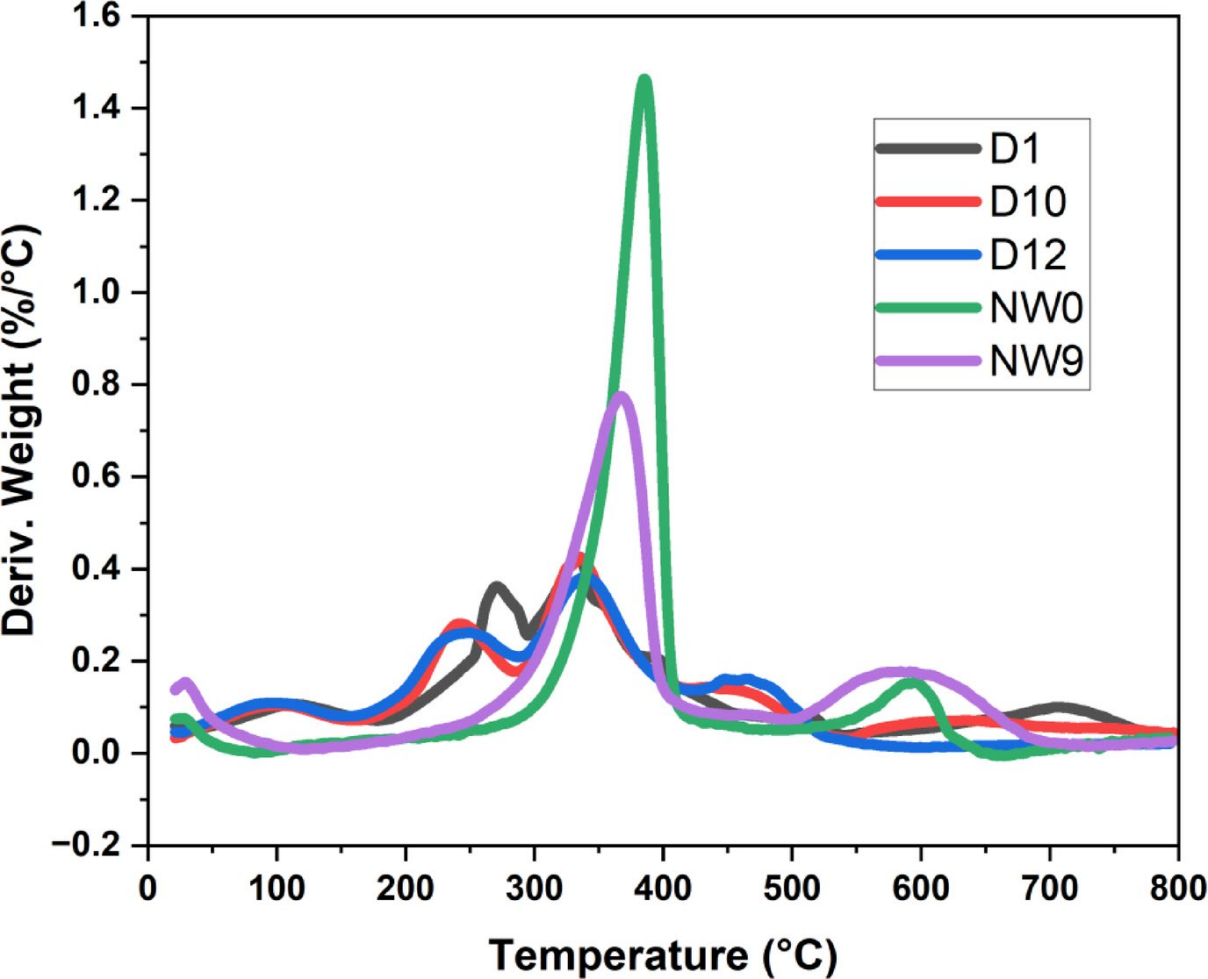
TGA and DTG curves for samples D1 (25:75% PVA/SC) film, D10 (PVA/SC/SH/Ag-NPs) film, D12 (PVA/SC/SH/Ag-NPs) film, NW0 (Blank non-woven cellulose), and NW9 (non-woven cellulose loaded by (PVA/SC/CUR/SH).

Figure 12, showed the TGA and DTG curves for samples D1 (25:75% PVA/SC) film, D10 (PVA/SC/SH/Ag-NPs) composite film, D12 (PVA/SC/SH/Ag-NPs) composite film, blank non-woven cellulose (NW0), and modified non-woven cellulose by PVA/SC/curcumin and shellac (NW9). The composite films showed four main stages for all film composites D1, D10 and D12 of TGA and DTG during the thermal decomposition process. It is helpful in figuring out the temperature at which a substance experiences a specific thermal event, like disintegration or combustion. The initial mass loss (ML) was about an average ML of 11.67% of D1, D10, and D12 composites at the same temperature ranges from 0 to 145 ºC, and DTG around 110 °C, this stage corresponds to moisture evaporation. The second degradation stage of D1, D10, and D12 composites exhibited an average ML of 23.49, 21.58, and 24.48% at about 145.32–300 °C and DTG around 267.91, 241.00 and 249.16 °C, respectively. This stage is attributed to vaporized water coordinated either chemically or physically on the composite surface, alongside dehydration of PVA/SC. The third major weight-loss stage of D1, D10, and D12 composites exhibited an average ML 26.66, 34.32 and 34.15% which occurred between 300 and 350, 300–415 °C and DTG around 335.59, 335.59 and 338.03 °C, respectively. This stage corresponds to deacetylation, depolymerization, and hydrocarbon backbone breakdown of the PVA/SC/SH/Ag-NPs composite. The fourth main weight-loss stage of D1, D10, and D12 composites exhibited an average ML 17.85, 27.64 and 18.24% which occurred between 350 and 445, 415–800 °C and DTG around 703.32, 450 and 475.83 °C, respectively. This stage involves breakdown of residual carbonaceous material into low molecular mass volatile compounds, char, and stable carbon compounds. Residual weight is linked to Ag-NPs at temperatures exceeding 700 ^o^C^53^.On the other hand, Fig. 12 show the thermal characteristics of the non-woven cellulose as in (NW0) and the modified non-woven cellulose by PVA/SC/CUR: SH as in (NW9) composites material which were examined using DTG and TGA in two decomposition stages. The first decomposition stage of non-woven cellulose NW0, and the modified non-woven cellulose (NW9) composites exhibited an initial ML of 80.40 and 68.92% at about 250–400 °C and DTG around 387.77 and 364.12 °C, respectively, this peak is distinguished by the elimination of moisture that has been absorbed from the cotton fiber. The main component of cotton is the hydrophilic cellulose, which has a strong affinity for moisture. Cotton fibers do not begin to degrade until they reach 200 degrees Celsius, according to the literature^17,70,71^. At around 315 °C, the temperature at which α-cellulose degrades, cotton fiber loses a significant amount of weight^72^. The second decomposition stage of NW0, and NW9 exhibited ML of 16.52 and 30.59% at about 450–650 and 400–800 °C and DTG around 598.95 and 586.72 °C, respectively. This stage reflects the breakdown of non-woven and modified PVA/SC/CUR-SH fibers.

TGA analysis revealed that adding PVA, SC, and CUR-SH enhanced the thermal stability. The modified cellulose decomposed 68.92% compared to 80% for untreated cellulose at 250–400 °C. Additionally, up to 543.87 °C, 35% of the modified cellulose remained stable, compared to 20% for untreated non-woven cellulose^69,72^. These indicate that the non-woven cellulose’s thermal stability was enhanced by the addition of PVA/SC/CUR-SH. These results align with improvements in mechanical properties like tensile and burst strength, and physical properties such as air permeability.

##### Water vapor transmission rate (WVTR)

**Fig. 13:**
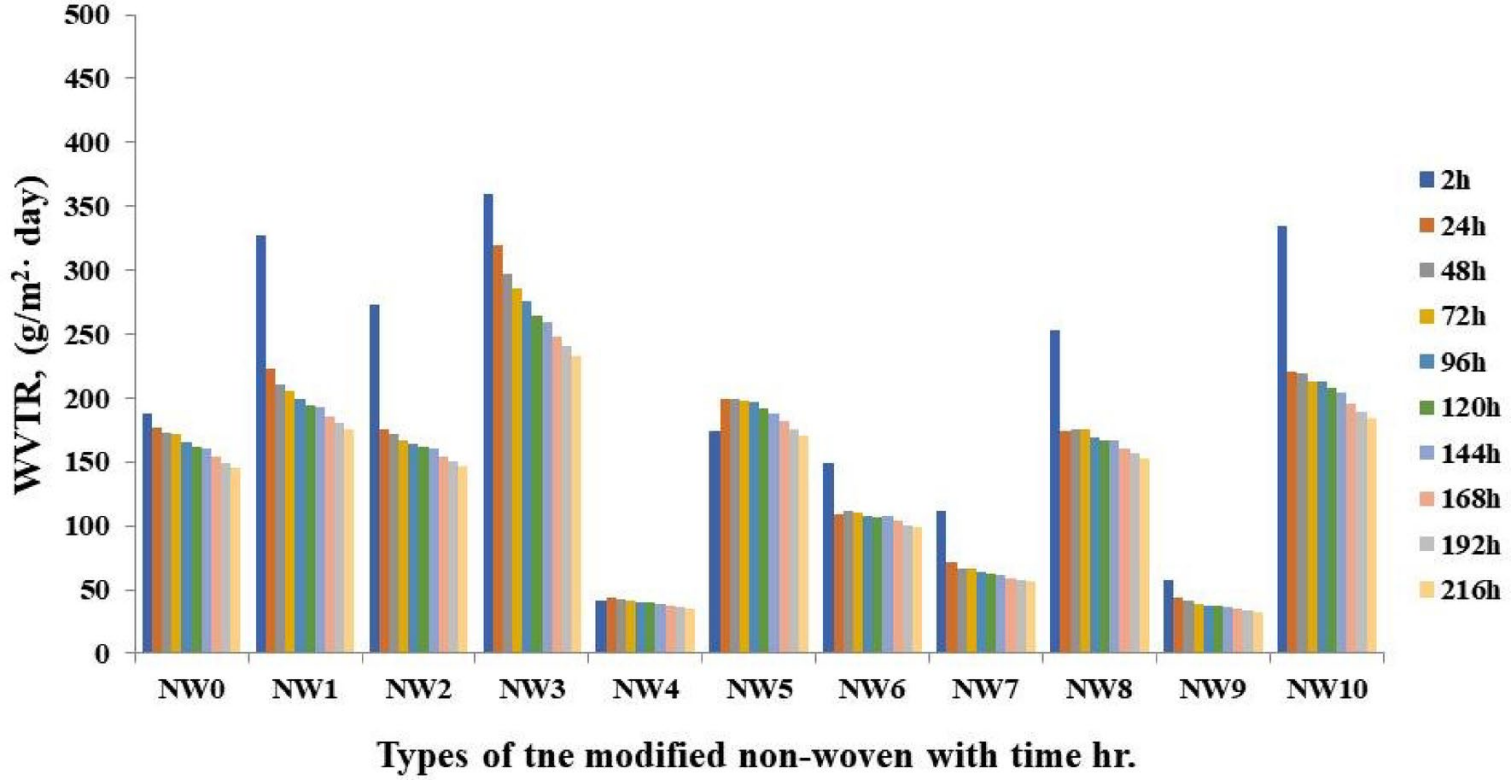
The water vapor transmission rate of PVA/SC/curcumin/shellac/Ag-NPs modified non-woven fabric.

The steady state rate at which water vapor permeates a film under particular temperature and relative humidity circumstances is known as the “water vapor transmission rate”. Lower values of WVTR indicate better moisture protection and are the standard metric used to compare the resistance of films to moisture transmission. As transmission rates are directly impacted by both temperature and humidity, results can only be meaningfully compared when measured under identical conditions. Without protective packaging, products would absorb and release moisture rapidly until they equilibrate with the surrounding relative humidity. This can lead to undesirable outcomes such as chewy food becoming hard and dry or crispy products turning soggy^73,74^. Among the modified non-woven films, the PVA/SC/0.075% curcumin/10% shellac formulation treated fabric (NW9) demonstrated the best WVTR, followed by PVA/SC/0.075% curcumin/3% Ag-NPs formulation treated fabric (NW4) and PVA/SC/10% shellac/2.5% Ag-NPs formulation treated fabric (NW7), as illustrated in Fig. 13.

The incorporated 0.075% curcumin/10% shellac formulation treated fabric (NW9) reduced the WVTR of the modified non-woven fabric’s by up to 77% compared to the untreated sample. This improvement is due to the smoother particle distribution, which effectively fills the gaps in the modified non-woven fabric network. The coated sheets exhibit lower water vapor transmission than the untreated ones, as curcumin and shellac form a dense, organized network on the fabric. This phenomenon can be explained by the cross-links formed within the coated modified non-woven fabric between the curcumin and shellac, which create a more complex pathway for water vapor to traverse, thereby reducing permeability. In contrast, employing curcumin alone, as in NW1, NW2, and NW3 (without shellac), results in higher WVTR values. This is attributed to the curcumin particles clumping together in the matrix, creating more open spaces rather than filling gaps. Without the presence of shellac particles to penetrate pores and fill the gaps in the modified non-woven fabric network, the resulting structure is less effective at resisting water vapor transmission^20^.

##### Measurement of contact angle

**Fig. 14:**
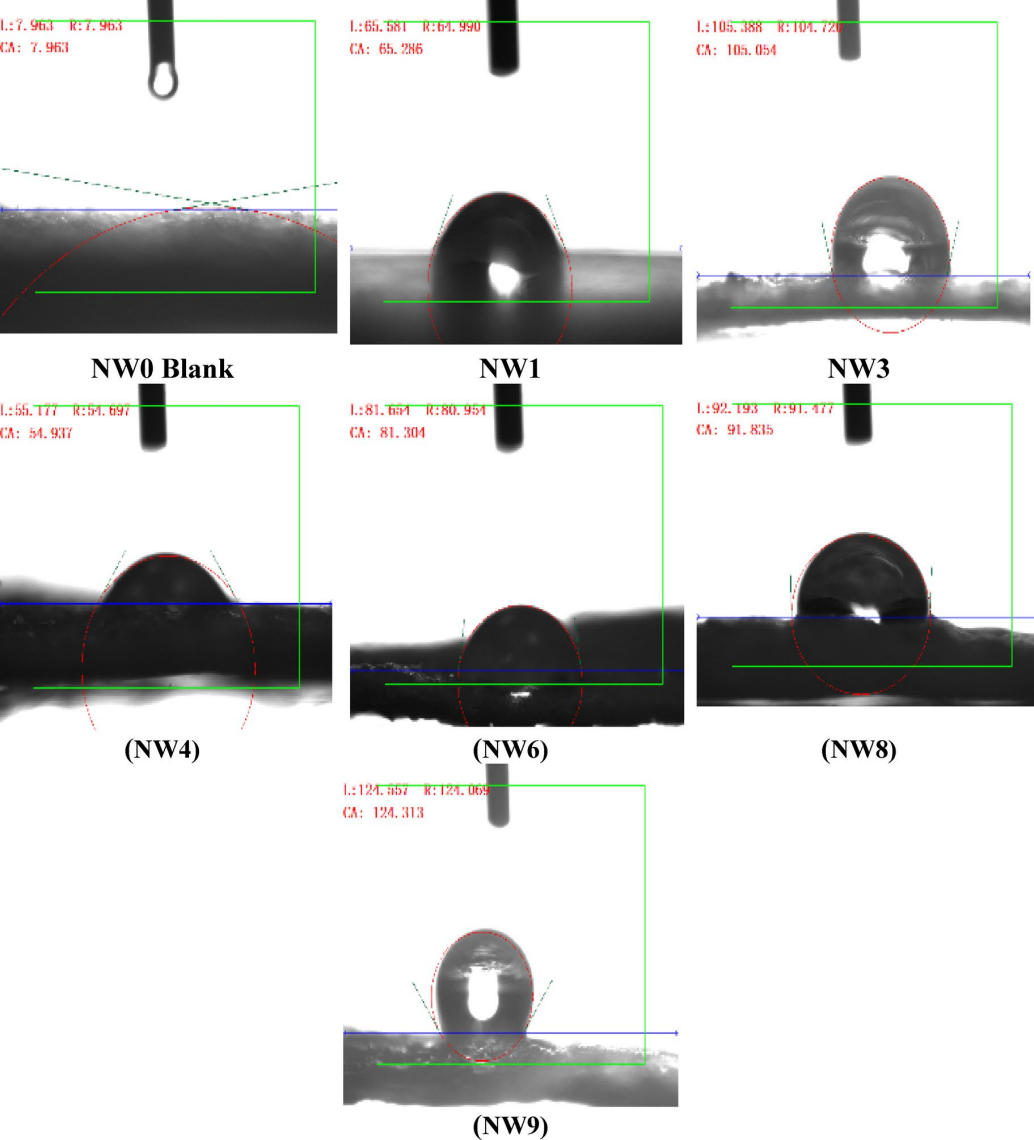
Films’ contact angles (wettability).

Water droplets were sprayed onto the composite non-woven, to measure the contact angle. The contact angle of a water droplet deposited on the surfaces of untreated non-woven sample could not be determined because the droplet was completely absorbed due to the super hydrophilicity of the untreated non-woven sample as in NW0 Blank, Fig. 14.

Figure 14 demonstrates that all treatments reduced the hydrophilicity of the non-woven fabric by increasing the contact angle. The increasing percent of the contact angles by the modified non-woven were measured as the following: (719.87%, 65.29°), (1219.28%, 105.05°), (584.88%, 54.54°), (921.02%, 81.30°), (1053.27%, 91.84°) and (1461.13%, 124.31°) corresponding to NW1, NW3, NW4, NW6, NW8 and NW9 respectively, compared to 7.96° of the untreated non-woven (NW0). This indicates a significant reduction in hydrophilicity after treatment with PVA/SC, with the modified non-woven samples becoming super-hydrophobic after the addition of PVA/SC/0.075% CUR and 0.075:10% of CUR/SH. The maximum hydrophobicity was observed with NW1 (PVA/SC) and NW9 (PVA/SC/CUR/SH) treatments, where contact angles increased by 1219.28% (105.05°) and 1461.13% (124.31°), respectively, compared to untreated non-woven.

The results indicate that the addition of PVA/SC, PVA/SC/0.075% CUR and 0.075:10% of CUR/SH to the pores of NW0 during the later stages of the reaction significantly enhanced the non-woven fabric’s resistance to water. This confirms and supports findings from other evaluations, including tensile strength, burst strength, air permeability, SEM, XRD and wettability measurements. These findings confirm that the methods used to mix and load PVA/SC, with or without curcumin, shellac or silver nanoparticles, onto the surface of non-woven cellulose were successfully and effective^12,55^.

##### Evaluation of toxicity

The Brine Shrimp Lethality Assay (BSLA) is a widely used method to evaluate the toxicity of various materials by measuring the mortality rate of brine shrimp (*A. salina*) nauplii after 24 h of exposure to different concentrations of the test materials. The results are often expressed as the LC_50_ value, which is the concentration of the material that causes 50% mortality in the brine shrimp population, as shown in Table 6.

**Table 6:** Toxicity evaluation of materials and composite films based on mortality rates of Brine shrimp (*A. salina*).

	Concentration of material extracts (mg/L)	Mortality after 24 h (%)	LC50 (ppm)
Casein	10	0	> 1000 ppm
	100	0.33	
	250	0.65	
	500	1.47	
	1000	5.61	
Shellac	10	0.53	847.2 ppm
	100	34.1	
	250	47.2	
	500	66.6	
	1000	89.7	
PVA	10	10	586.3 ppm
	100	18.2	
	250	27.2	
	500	33.3	
	1000	56.6	
Curcumin	10	0	> 1000 ppm
	100	1.33	
	250	11.3	
	500	22.3	
	1000	35.1	
D12	10	10.3	687.6 ppm
	100	23.33	
	250	33.6	
	500	51.7	
	1000	69.8	
L.S.D at 0.5%		2.76	

Casein shows minimal toxicity to brine shrimp, even at high concentrations. At 1000 mg/L, casein recorded a ratio of 5.61% mortality (indicating very low toxicity). This suggests that casein is a biocompatible material with low environmental risk. Shellac exhibits concentration-dependent toxicity. At lower concentrations, such as 10 mg/L, shellac caused 0.53% mortality (relatively safe). However, at higher concentrations (1000 mg/L), the mortality ratio increased significantly to 89.7%. However, the LC_50_ value indicates moderate toxicity compared to other materials. PVA shows moderate toxicity, with mortality increasing steadily as concentration rises. The LC_50_ value reveals that PVA is more toxic than casein but less toxic than shellac. Curcumin is relatively non-toxic at lower concentrations but shows moderate toxicity at higher levels. Its LC_50_ value indicates low overall toxicity, comparable to casein.

The composite film D12 (PVA/SC/SH/Ag-NPs) exhibited an LC_50_ value between those of PVA and shellac. This suggests that the combination of materials in the composite influences its toxicity. At a concentration of 10 mg/L, the applied film recorded a mortality ratio of 10.3%, indicating low toxicity. However at 1000 mg/L, the mortality ratio increased to 69.8%, which highlights the importance of avoiding high concentrations in practical applications, as shown in Table 6.

##### The antibacterial properties of treated non-woven cotton fabric

**Fig. 15:**
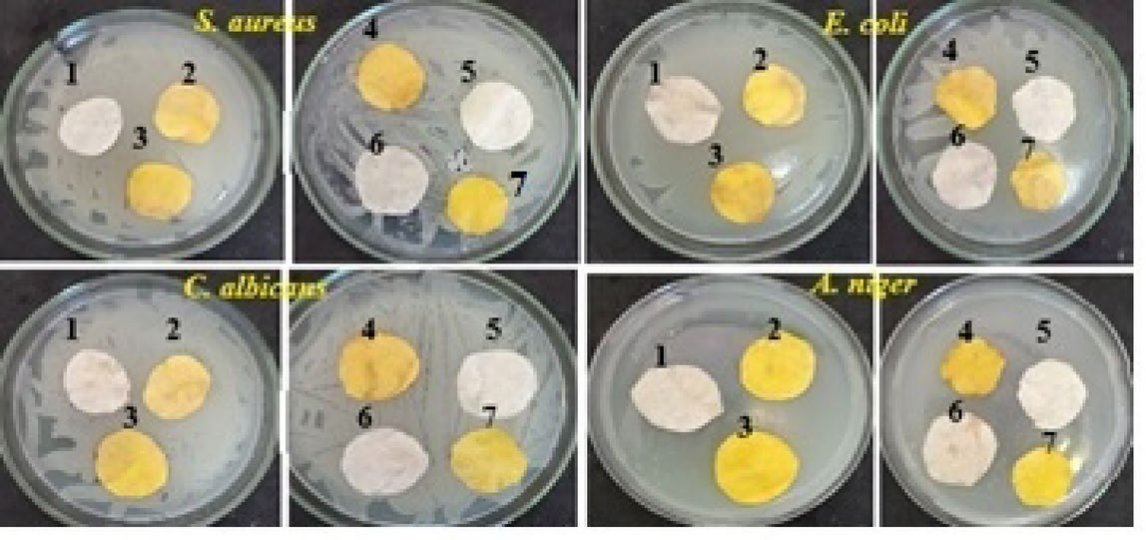
The antimicrobial activity of the treated fabric samples NW4 (1) -NW10 (7) against Gram-positive bacteria (*S. aureus*), Gram-negative bacteria (*E. coli*), Yeast (*C. albicans*) and fungi (*A. niger*).

Despite the antibacterial properties of curcumin^39,75^, the PVA/SC/curcumin formulation treated sample does not exhibit antimicrobial properties as shown in Table 7. The curcumin concentration in the PVA/SC/curcumin formulation was insufficient to impart antibacterial properties to the treated NWC fabric samples. However, increasing the curcumin concentration inside the PVA/SC/curcumin formulation leads to cracked films (Fig. 15D and E). Thus, it is necessary to incorporate additional bio-additive such as shellac or Ag-NPs, into the PVA/SC/curcumin formulation. These bio-additives enhance the antimicrobial activity without compromising the coated layer of the treated fabric.

The antibacterial properties of the PVA/SC formulations treated samples are listed in Table 7 and designated by NW1-NW10. It is clear that: (a) all the treated samples loaded solely with curcumin failed to exhibit antimicrobial activities against Gram-positive bacteria, *Staphylococcus aureus* and Gram-negative bacteria, *Escherichia coli*, and (b) The inclusion of either shellac or Ag-NPs in the formulations significantly improved the antibacterial properties of treated fabric against these bacterial strains^37^.

Moreover, Fig. 15 shows the antimicrobial activities of the treated samples NW1-NW10 against Gram-positive bacteria: *Staphylococcus aureus*, Gram-negative bacteria: *Escherichia coli*, pathogenic yeast: *Candida albicans*, and filamentous fungus: *Aspergillus niger*. Although no inhibition zones were observed in any treated samples, microbial growth was also absent. This phenomenon be attributed to the antimicrobial properties of Ag-NPs and shellac^37,39,75^, along with the strong binding of the bio-additives molecules to the PVA/SC blend polymeric structure. This binding prevents bio-additive molecules from dissipating out of the PVA/SC film structure to form inhibition zone against the attacking microbial cells. Further analysis of the SEM images confirms the presence of silver nanoparticles embedded within the polymeric structure. The interaction between PVA/SC and silver nanoparticles or curcumin/shellac with silver nanoparticles leads to the formation of macromolecular complexes, enhancing the antimicrobial properties of the treated fabric.

**Table 7:** Antibacterial properties of the PVA/SC formulation treated non-woven cotton fabric.

Formulation type	Observations	
	Gram-positive	Gram-negative
NW1	Bacterial growth	Bacterial growth
NW2	Bacterial growth	Bacterial growth
NW3	Bacterial growth	Bacterial growth
NW4	No bacterial growth	No bacterial growth
NW5	No bacterial growth	No bacterial growth
NW6	No bacterial growth	No bacterial growth
NW7	No bacterial growth	No bacterial growth
NW8	No bacterial growth	No bacterial growth
NW9	No bacterial growth	No bacterial growth
NW10	No bacterial growth	No bacterial growth

#### Diagram for the PVA/SC film formation and the treated non-woven fabric

Schema 2 illustrates the formation process and suggested mechanism for PVA/SC film and treated non-woven fabric, highlighting their novel potential as packaging materials. These materials are created by treating non-woven cotton fabric samples with various formulations containing CS, PVA, and different antimicrobial additives, such as curcumin, shellac, silver nanoparticles, or their binary admixtures. Casein is relatively hydrophobic, the matter that limiting its solubility in water. Alternatively, to enhance the solubility, casein can be transformed into sodium caseinate, which is commonly used. In this study, sodium caseinate was acidified with acetic acid to replace the Na^+^ ions with H^+^. This transformation increases hydrophobicity, making the casein matrix more suitable as a potential packaging material (Schema 2).

## Conclusions

- The ideal conditions for preparing crosslinked PVA/SC film with superior performance properties are: PVA/SC weight ratio, 25/75 respectively; Acetic acid concentration, 0.25%; and immersion time, 10 min.
- Toxicity evaluation revealed that, casein exhibits minimal toxicity, even at high concentrations. At 1000 mg/L, casein showed only 5.61% mortality, confirming its biocompatibility and low environmental risk. Shellac is relatively safe at lower concentrations, with 0.53% mortality at 10 mg/L. However, its toxicity increases significantly at higher concentrations (89.7% mortality at 1000 mg/L). PVA shows moderate toxicity, with a steady increase in mortality as concentration rises. Curcumin is relatively non-toxic at lower concentrations and exhibits low overall toxicity, similar to casein. The composite film D12 (PVA/SC/SH/Ag-NPs) demonstrated a 10.3% mortality ratio at 10 mg/L (low toxicity).

FTIR analysis proved the chemical structure of the prepared PVA/SC film, while SEM analysis confirmed the morphology of treated fabric samples. The PVA/SC film containing curcumin/shellac (weight ratio of 0.075:10, respectively) exhibited the best performance properties among all formulations.

Applying the PVA/SC/curcumin/shellac formulation to non-woven cotton fabric results in: (a) an increasing in gel fraction, along with a reduction in swelling properties, (b) improved tensile strength, Young’s and burst strength, paired with a decrease in air permeability, (c) enhanced the water vapor transmission rate compared to the other treated fabric samples, (d) Maximum hydrophobicity, with contact angle increases of 1219.28% (105.05°) for NW1 (PVA/SC) and 1461.13% (124.31°) for NW9 (PVA/SC/CUR/SH), compared to untreated non-woven fabric, (e) Successful integration of PVA/SC or PVA/SC/Cur/SH into non-woven cellulose, as evidenced by the X-ray, where decreased crystallinity confirmed effective surface reactions. (f) TGA indicate that the non-woven cellulose’s thermal stability was enhanced by the addition of PVA/SC/CUR-SH, and (g) an enhancement in the antimicrobial activities of the treated fabric against Gram-positive bacteria *Staphylococcus aureus*, Gram-negative bacteria: *Escherichia coli*, pathogenic yeast: *Candida albicans*, and filamentous fungus: *Aspergillus niger*.

These findings strongly support the potential application of treated fabric with PVA/SC/curcumin/shellac formulation as a sustainable, antimicrobial packaging material.

## Data Availability

All the data generated in this research work has been included within this manuscript."All data generated or analysed during this study are included in this published article".
